# Supplementary material for: Promoting antigen escape from dendritic cell endosomes potentiates anti-tumoral immunity
Source: Cell Rep Med. 2022 Feb 25;3(3):100534. doi: 10.1016/j.xcrm.2022.100534 (PMC9040180; doi:10.1016/j.xcrm.2022.100534)
Supplement: Document S1. Figures S1–S8 [file mmc1.pdf]

Cell Reports Medicine, Volume *3*

Supplemental information

Promoting antigen escape from dendritic cell endosomes potentiates anti-tumoral immunity

Jean-Pierre Bikorimana, Natasha Salame, Simon Beaudoin, Mohammad Balood, Théo Crosson, Jamilah Abusarah, Sebastien Talbot, Raimar Löbenberg, Sebastien Plouffe, and Moutih Rafei

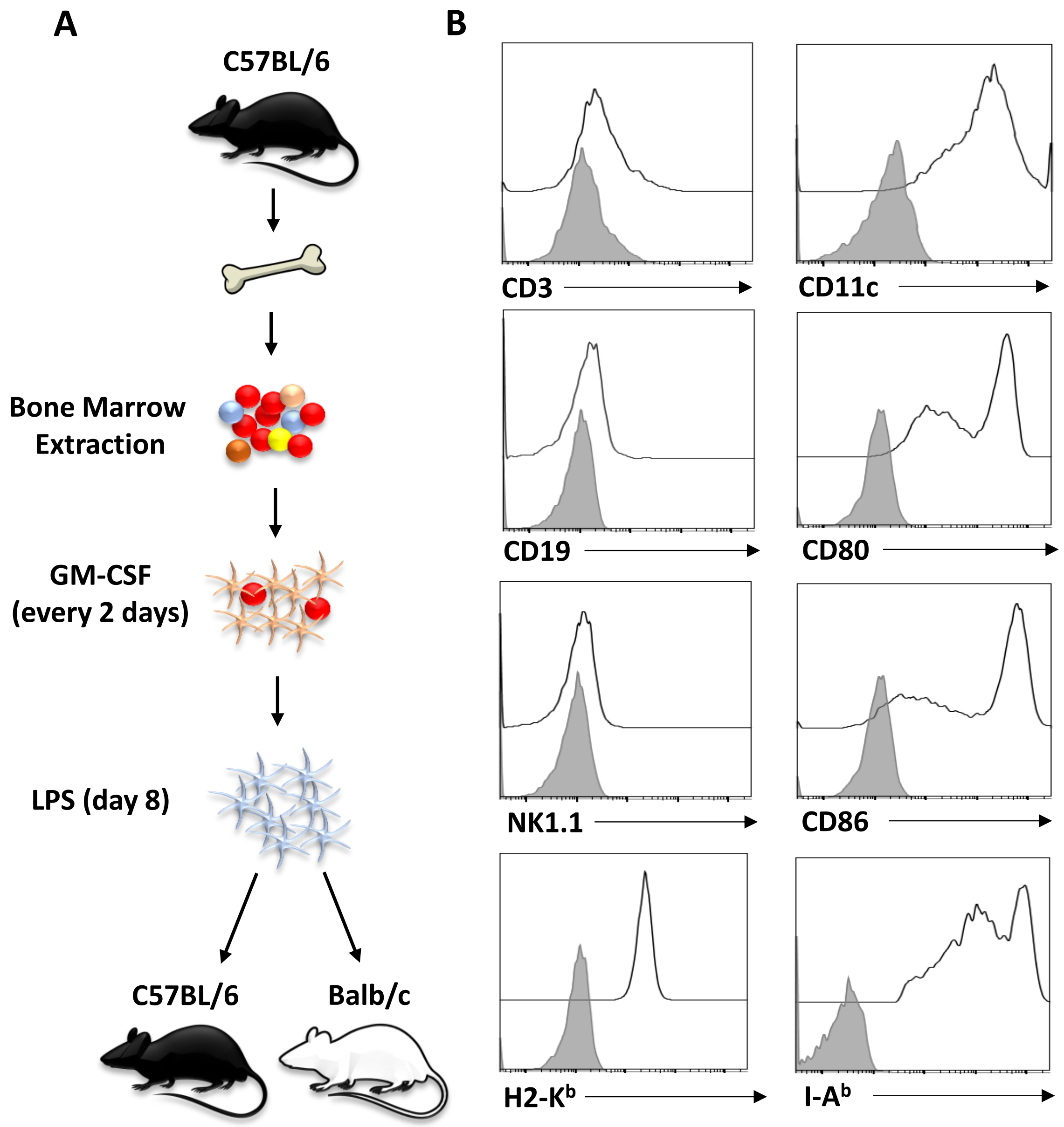

Supplementary figure 1: Generation and characterisation of BM-derived DC for vaccination. A) To generate BM-derived mature DCs, femur and tibias of female C57BL/6 or Balb/c mice are flushed to collect total nucleated cells. Cells are then plated for 8 days with recombinant GM-CSF (10 ng/ml) and replaced every 2 days. LPS is added on day 9 to trigger DC maturation prior to antigen pulsing. **B)** Representative flow-cytometry analysis of mature DC phenotype. No T cells, B cells or NK cells were detected at day 9. More than 80% of *ex vivo* generated mature DCs expressed $CD11c^{+}$, $CD80^{+}$, $CD86^{+}$, and $I\text{-}A^{b+}$. Related to figures 2, 4, 5 and 6.

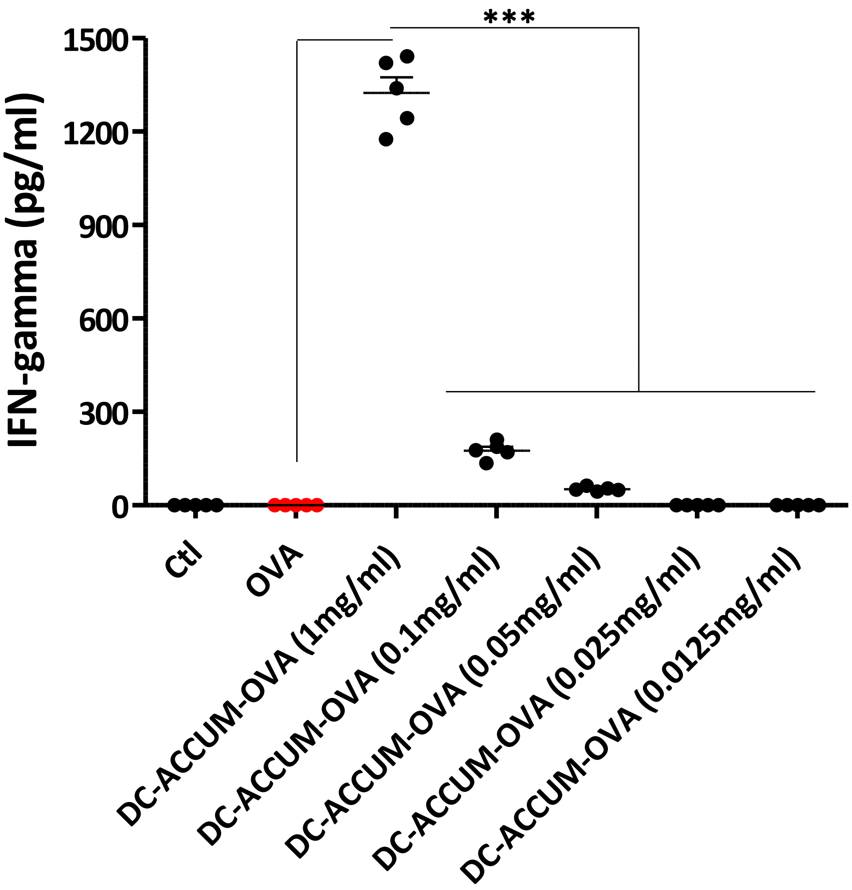

Supplementary figure 2: Quantification of IFN-gamma production by OT-II T cells. An antigen presentation assay was conducted using OT-II-derived CD4 T cells co-cultured with decreasing concentration of aOVA-pulsed mature DCs versus nOVA-pulsed mature DCs (shown in red). Three days following the co-culture, IFN-gamma was quantified using a commercial ELISA. For this panel, n=5/group with ***P<0.001. Related to figure 2.

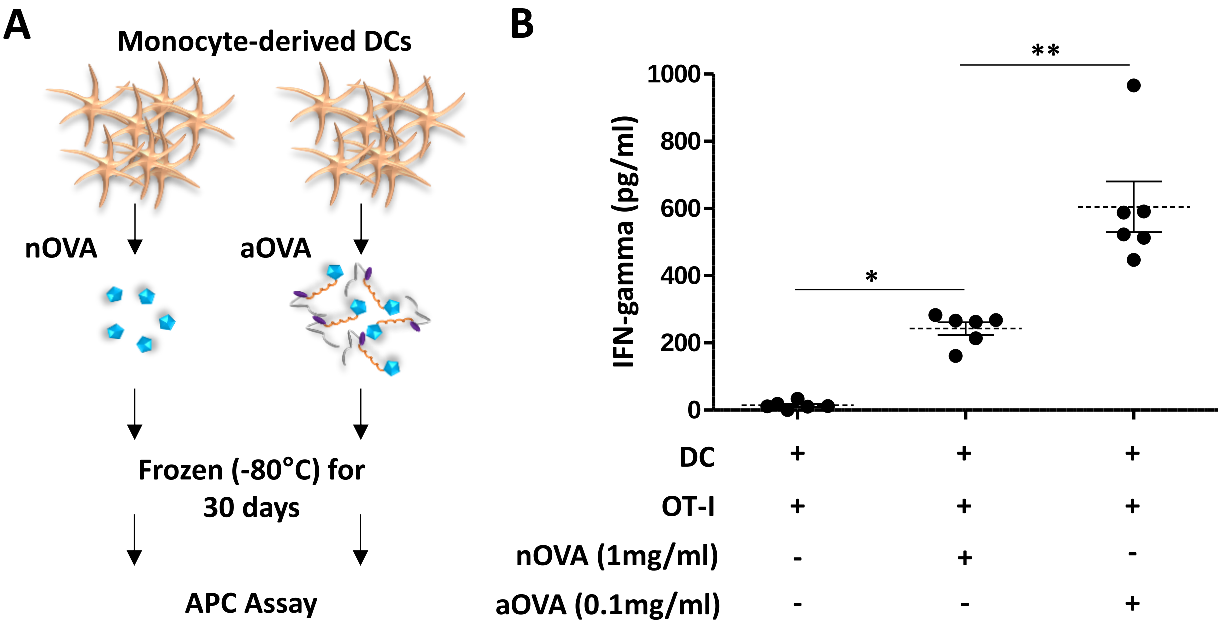

Supplementary figure 3: Frozen aOVA-pulsed mature DCs retain their potency to activate CD8 T cells. A) Representative cartoon of the experimental setting. **B)** Antigen presentation assay using frozen mature DCs originally pulsed with nOVA or aOVA. For this panel, n=6/group with *P<0.05 and **P<0.01. Related to figure 2.

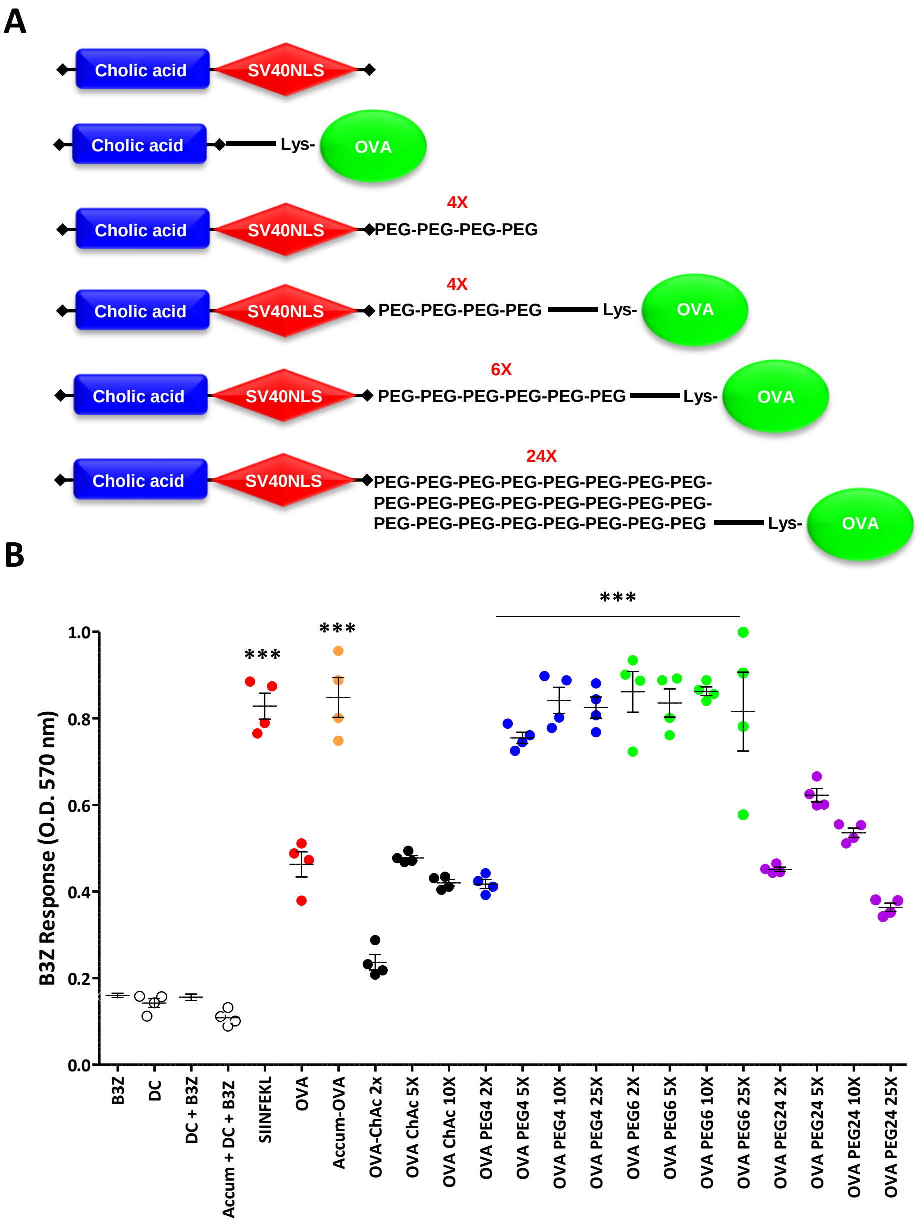

Supplementary figure 4: Testing the effect of various aOVA variants on the efficacy of antigen presentation by DCs. A) Representative cartoon of the various variants. The numbers 4X, 6X and 24X refers to the number of PEG molecules par Accum construct. The Accum-OVA is represented in the three-cartoon containing Cholic acid, SV40NLS, PEG and OVA. **B)** Response quantification using the SIINFEKL-specific B3Z cell line co-cultured with mature DCs treated with the different variants. The numbers 2X, 5X, 10X, and 25X refers to the number of Accum molecule per OVA. For this panel, n=5/group with ***P<0.001 when compared to the nOVA group. Related to figure 2.

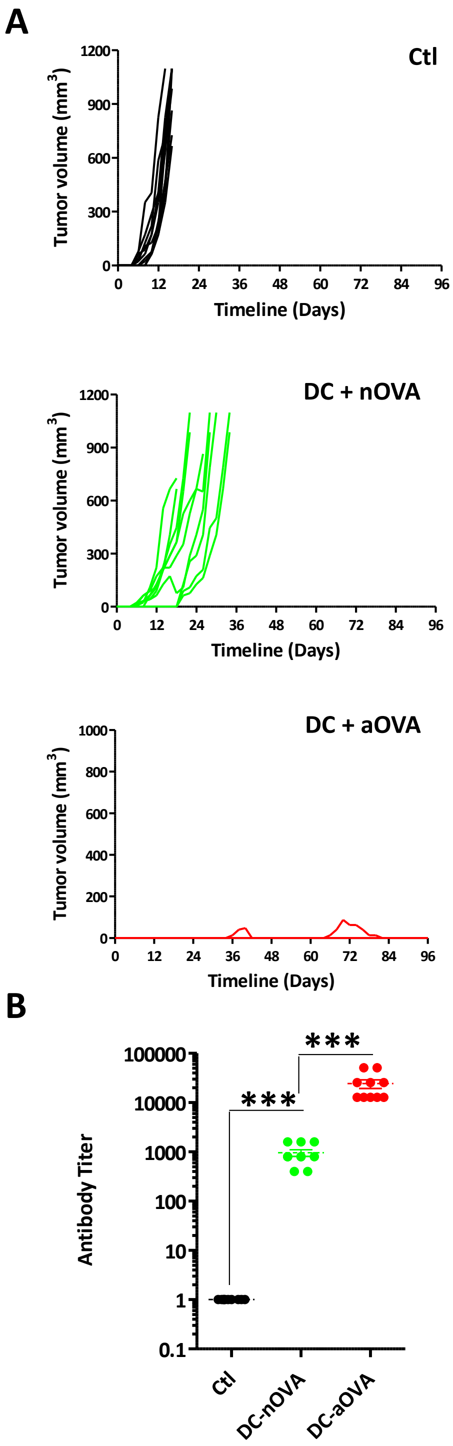

Supplementary figure 5: Individual tumor measurements and antibody tiers form the prophylactic vaccination experiment. A) Individual tumor measurements for EG.7 tumor growth in control mice (in black), OVA-pulsed mature DC-vaccinated mice (green) or Accum-OVA-pulsed mature DCs (red). **B)** Assessment of anti-OVA IgG titer by ELISA. For this experiment, n=10/group with ***P<0.001. Related to figure 4.

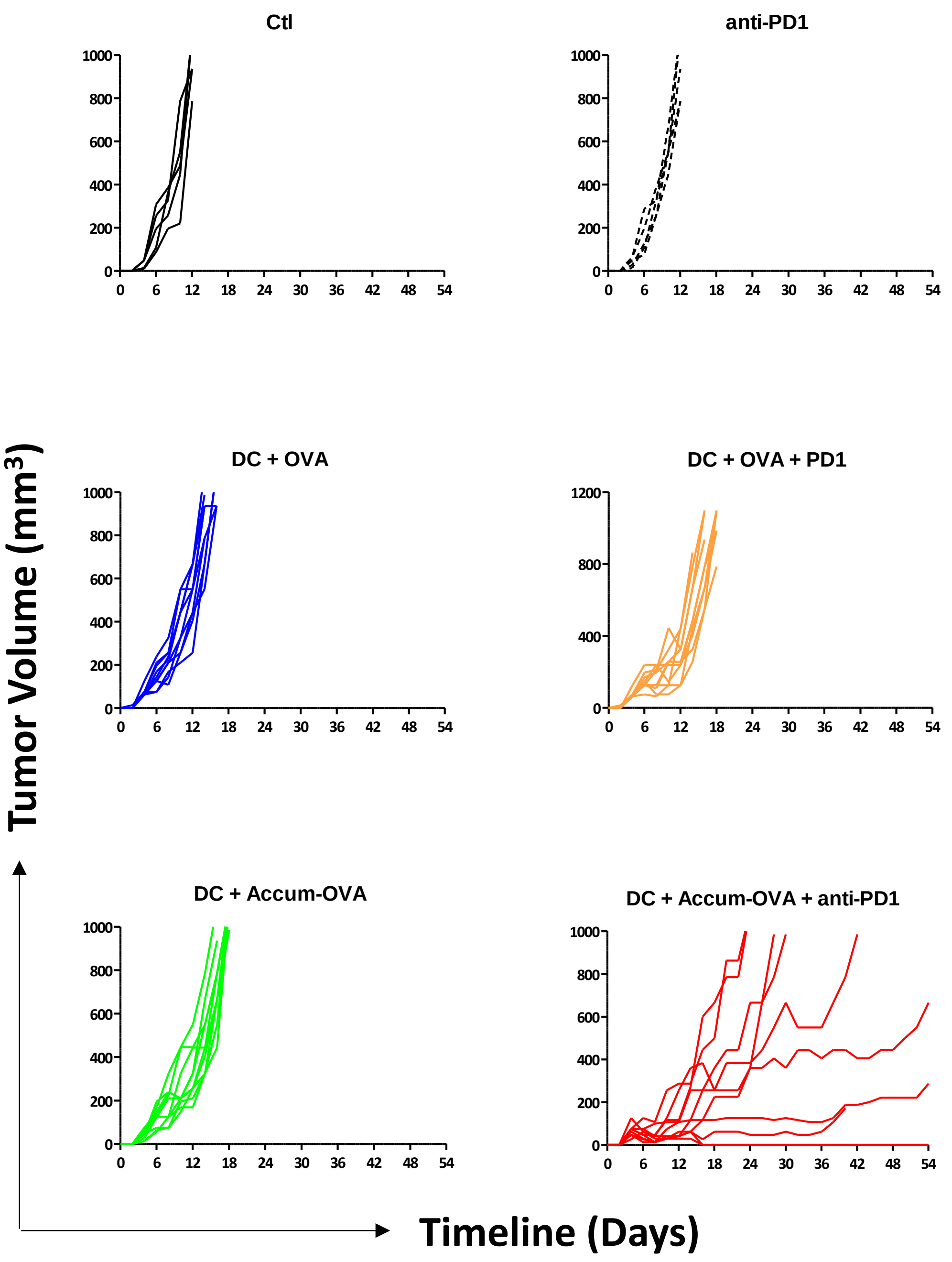

Supplementary figure 6: Individual tumor measurements from the syngeneic therapeutic vaccination study targeting EG.7 lymphoma. Individual tumor measurements for EG.7 tumor growth in control mice (in black), anti-PD-1-injected mice (black dotted lines), OVA-pulsed mature DC-vaccinated mice (blue), OVA-pulsed mature DC-vaccinated mice receiving anti-PD-1(orange), Accum-OVA-pulsed mature DCs (green), or Accum-OVA-pulsed mature DCs receiving anti-PD-1 (red). For this experiment, n=10/group. Related to figure 5.

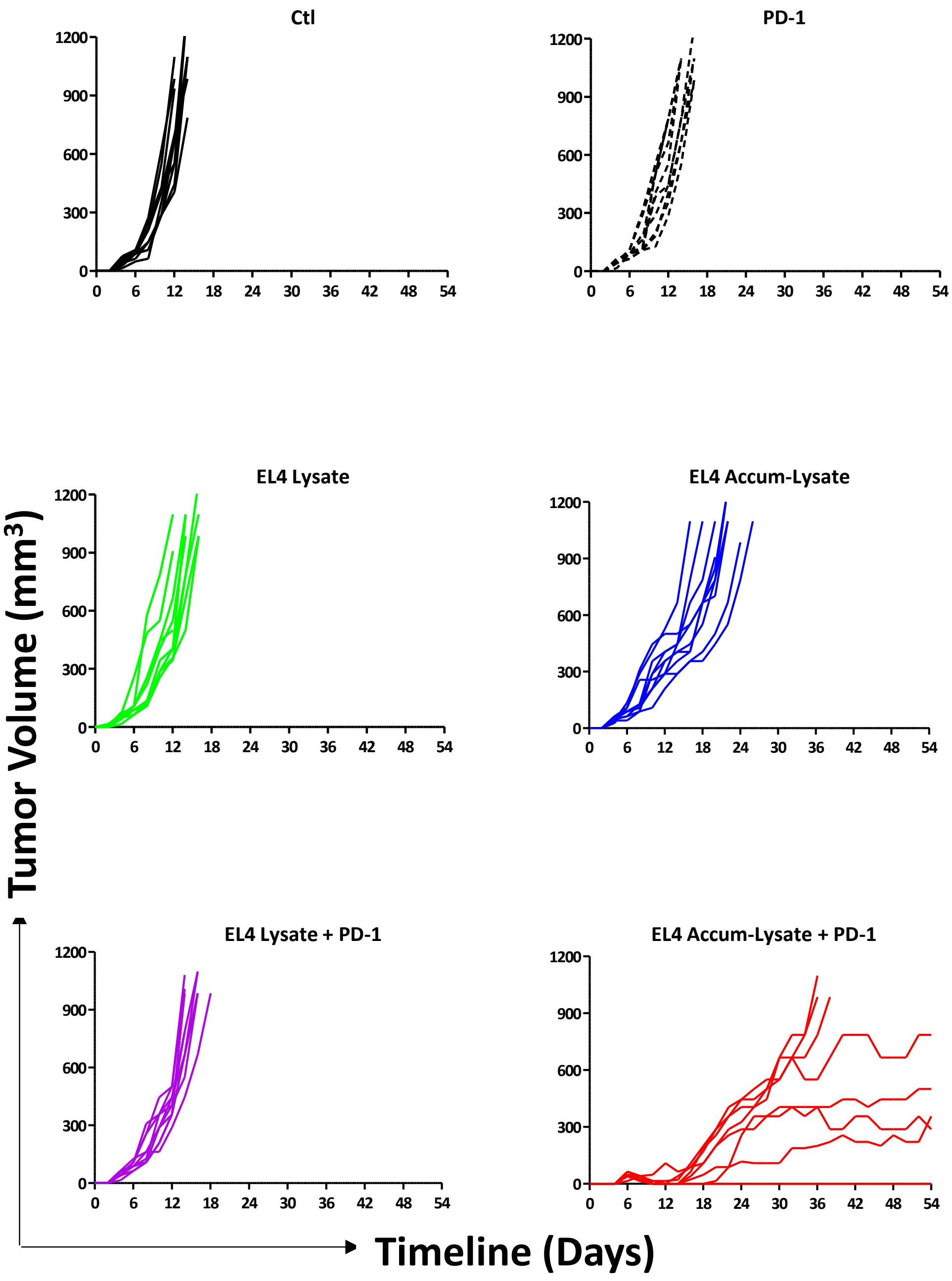

Supplementary figure 7: Individual tumor measurements from the allogeneic therapeutic vaccination targeting EL4 lymphoma. Individual tumor measurements for EL4 tumor growth in control mice (in black), anti-PD-1- injected mice (black dotted lines), EL4 lysate-pulsed mature DC-vaccinated mice (green), EL4 Accum-lysate-pulsed mature DC-vaccinated mice (blue), EL4 lysate-pulsed mature DC-vaccinated mice receiving anti-PD-1 (purple), or EL4 Accum-lysate-pulsed mature DC-vaccinated mice receiving anti-PD-1 (red). For this experiment, n=10/group. Related to figure 6.

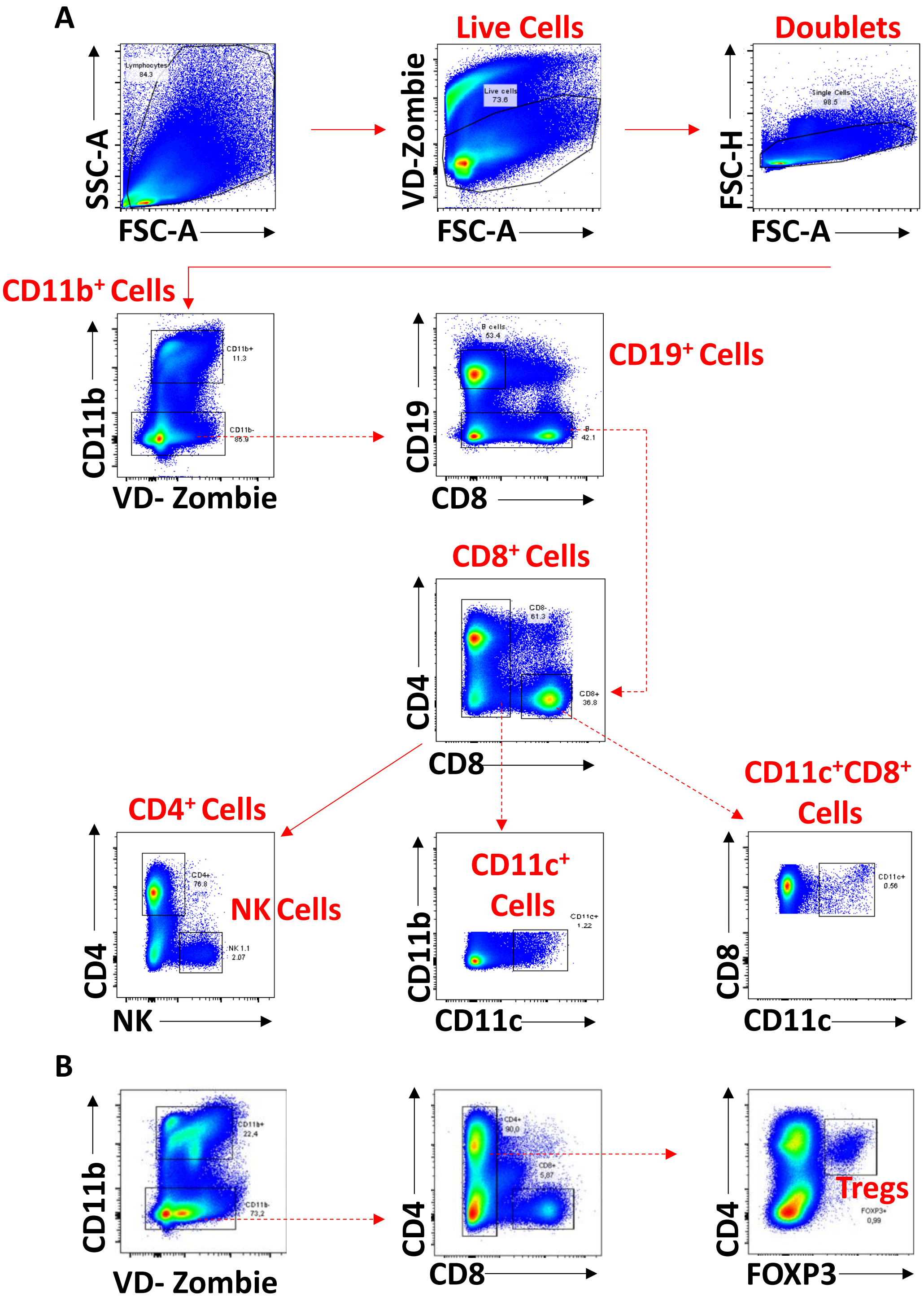

Supplementary figure 8: Gating strategies used for the TIL study. A) A representative gating strategy to demonstrate how various immune cells were identified. **B)** A representative gating strategy used to identify Tregs. Related to figure 6.
