# Supplementary material for: Promoting antigen escape from dendritic cell endosomes potentiates anti-tumoral immunity
Source: Cell Rep Med. 2022 Feb 25;3(3):100534. doi: 10.1016/j.xcrm.2022.100534 (PMC9040180; doi:10.1016/j.xcrm.2022.100534)
Supplement: Document S2. Article plus supplemental information [file mmc2.pdf]

Cell Reports
Medicine

Article

Promoting antigen escape from dendritic cell endosomes potentiates anti-tumoral immunity

Graphical abstract

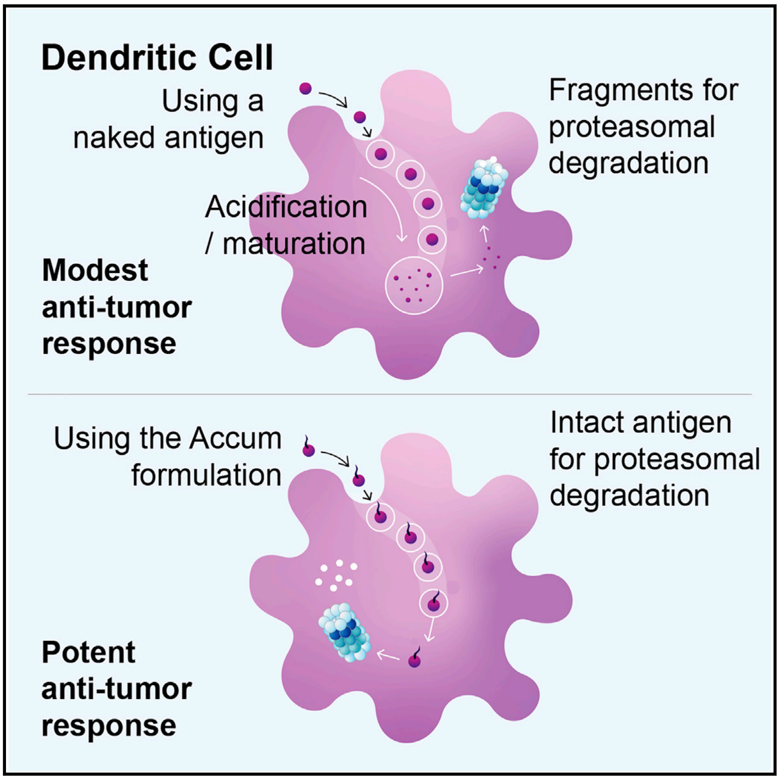

Highlights

- Accum-linked antigen enhances antigen processing and presentation
- Pulsed dendritic cells elicit potent effector T cell responses
- Therapeutic vaccination using allogeneic DCs controls pre-established tumors
- The vaccine boosts tumor-infiltrating lymphocytes and increases the CD8/Treg ratio

Authors

Jean-Pierre Bikorimana, Natasha Salame, Simon Beaudoin, ..., Raimar Löbenberg, Sebastien Plouffe, Moutih Rafei

Correspondence

moutih.rafei.1@umontreal.ca

In brief

Bikorimana et al. present dendritic cells pulsed with Accum-antigen formulation. The resulting optimized degradation pathway for antigen cross-presentation leads to potent anti-tumoral response and triggers endogenous immune effector cells to attack established tumors. These attributes highlight the use of this technology in recycling past DC vaccines or designing future ones.

Bikorimana et al., 2022, Cell Reports Medicine *3*, 100534
March 15, 2022 © 2022 The Author(s).
https://doi.org/10.1016/j.xcrm.2022.100534

CellPress

Cell Reports Medicine

CellPress
OPEN ACCESS

Article

Promoting antigen escape from dendritic cell endosomes potentiates anti-tumoral immunity

Jean-Pierre Bikorimana,[1] Natasha Salame,[2] Simon Beaudoin,[3] Mohammad Balood,[4] Théo Crosson,[4] Jamilah Abusarah,[4] Sebastien Talbot,[4] Raimar Löbenberg,[5] Sebastien Plouffe,[3] and Moutih Rafei[1,4,6,7,*]
[1]Department of Microbiology, Infectiology and Immunology, Université de Montréal, Montréal, QC, Canada
[2]Department of Biomedical Sciences, Université de Montréal, Montréal, QC, Canada
[3]Research and Development Branch, Defence Therapeutics Inc., Vancouver, BC, Canada
[4]Department of Pharmacology and Physiology, Université de Montréal, Montréal, QC, Canada
[5]Department of Pharmacy and Pharmaceutical Sciences, University of Alberta, Edmonton, AB, Canada
[6]Molecular Biology Program, Université de Montréal, Montréal, QC, Canada
[7]Lead contact
*Correspondence: moutih.rafei.1@umontreal.ca
https://doi.org/10.1016/j.xcrm.2022.100534

SUMMARY

The cross-presenting capacity of dendritic cells (DCs) can be limited by non-specific degradation during endosome maturation. To bypass this limitation, we present in this study a new Accum-based formulation designed to promote endosome-to-cytosol escape. Treatment of primary DCs with Accum linked to the xenoantigen ovalbumin (OVA) triggers endosomal damages and enhances protein processing. Despite multiple challenges using ascending doses of tumor cells, DC prophylactic vaccination results in complete protection due to increased levels of effector CD4 and CD8 T cells as well as high production of pro-inflammatory mediators. When combined with anti-PD-1, therapeutic vaccination using both syngeneic and allogeneic Accum-OVA-pulsed DCs triggers potent anti-tumoral responses. The net outcome culminates in increased CD11c, CD8, and NK infiltration along with a high CD8/Treg ratio. These highly favorable therapeutic effects highlight the promising potential of Accum as a distinct and potent technology platform suitable for the design of next generation cell cancer vaccines.

INTRODUCTION

Anti-tumoral immunity relies on antigen cross-presentation by dendritic cells (DCs).[1,2] For this process to occur, soluble antigens must be first engulfed and sorted into endosomes, whose main function is to initiate limited degradation of captured antigens by lysosomal proteases so they can be exported to the cytosol as large polypeptide fragments for further processing by the proteasomal complex.[3] The generated eight to nine amino acid peptides are then loaded onto cell surface major histocompatibility complex (MHC) class I molecules to activate responding CD8 T cells.[3] Since the proteolytic activity of endosomal proteases is optimal at acidic pH, maturing endosomes undergo progressive acidification through the recruitment of several V-ATPase subunits.[4–9] Interestingly, some DC subsets such as $CD8^+$DCs in mice (described as the equivalent to $CD141^+XCR1^+$ in humans[10]), have developed specific means to minimize endocytic acidification in order to protect captured antigens from uncontrolled or exacerbated degradation.[8] One of these mechanisms consists of assembling the NADPH oxidase (NOX)2 in $CD8^+$DC endosomes, which would lead to reactive oxygen species production within endosomal lumen to prevent acidification.[4–9] This explains the distinct cross-presentation abilities of $CD8^+$DCs compared with monocyte-derived $CD8^-$DCs.[8] Unfortunately however, the use of these cross-presenting DCs for the development of an *ex vivo* DC vaccine pulsed with an antigenic preparation is difficult to achieve with their limited number in peripheral blood of mice and humans.[11] Besides, the alternative preparation of cross-presenting DCs for vaccine applications using the induced pluripotent stem technology is both costly and time-consuming.[11] Therefore, novel strategies must be designed to tightly control or modulate endosomal degradation in monocyte-derived DCs as a means to avoid damaging/destroying antigen fragments important for the generation of immunogenic peptides endowed with the capacity to elicit effective anti-tumoral immunity.

Besides its negative impact on vaccination, degradation of proteins by endo-lysosomal organelles has been long recognized as a major deterrent to various therapeutic treatments, including antibody-drug conjugates.[12] Of the many attempts to optimize intracellular drug delivery, Beaudoin et al. described a novel formulation technology whereby a given therapeutic antibody conjugated to an Accum moiety (composed of a cholic acid [ChA] coupled to a nuclear localization sequence [NLS]) accumulates efficiently in the cytosol of target cells by disrupting endosomal membranes.[12] We thus elected to investigate whether applying such strategy to antigen cross-presentation improves the immune-therapeutic potency of *ex vivo* developed

This is an open access article under the CC BY license (http://creativecommons.org/licenses/by/4.0/).

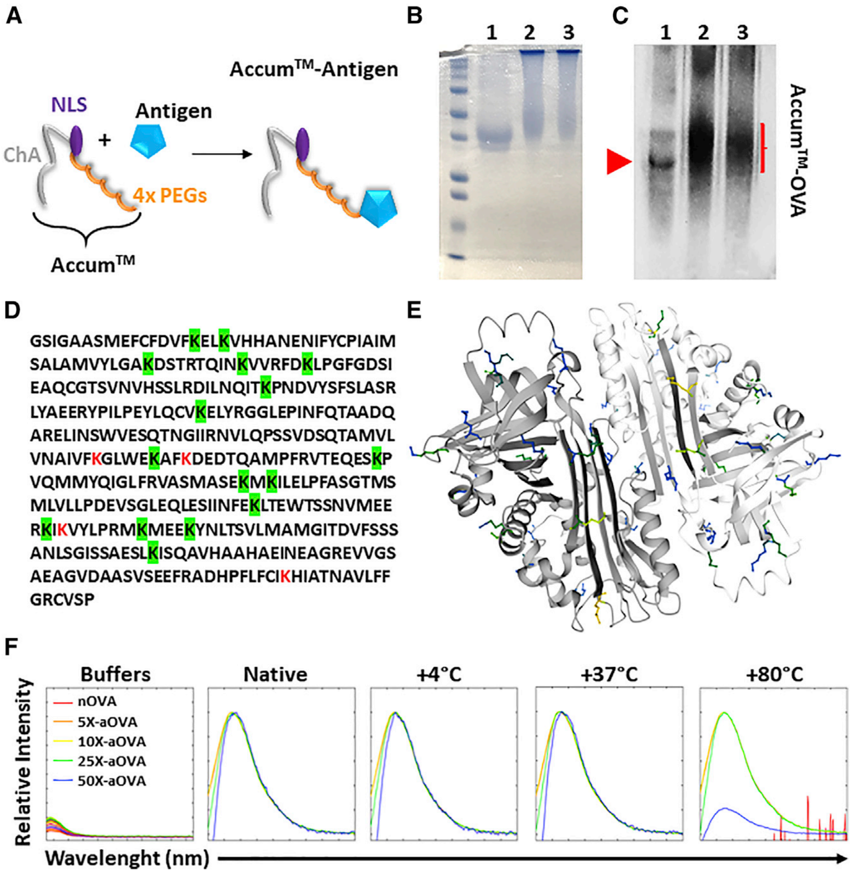

Figure 1. Biochemical characterization of the Accum-antigen formulation

(A) Schematic diagram representing covalent binding of a given antigen to the Accum moiety (ChA, NLS and 4x PEGs).

(B) A representative Coomassie blue staining displaying OVA (line 1), aOVA at a ratio of 25X (line 2), and aOVA at a ratio of 50X (line 3).

(C) A representative western blot of the gel shown in (B).

(D) The amino acid sequence of chicken OVA. Lysine residues that are predicted to be accessible for Accum linking (>50%) are highlighted in green. The three weakly accessible residues are shown in red.

(E) A ribbon structure of the OVA protein with lysine residues that are predicted to be highly (in blue), moderately (green), or poorly (yellow) accessible lysine residues.

(F) ITF analysis of nOVA or aOVA at various Accum to OVA ratios in response to thermal stress. The experiment presented in (B) was repeated at least 10 times, whereas (F) is a representative study of two independent repeats.

CD8^{-}DCs. Compared with naked (n) ovalbumin (OVA), DCs pulsed with Accum (a)OVA elicit potent CD4 and CD8 T cell activation. The net outcome culminates into effective anti-tumoral responses even when the formulation is conjugated to total tumor lysate instead of a single defined antigen. We also demonstrate how this strategy can be easily adapted to allogeneic DCs, which would pave the path for the future development of universal therapeutic vaccines.

RESULTS

Biochemical characterization of aOVA bioconjugate

To generate the aOVA final product, a chemical reaction linking an Accum moiety (consisting of ChA, NLS, and four x PEG molecules) to lysine residues of nOVA was performed (Figure 1A). This led to changes in the molecular weight of the protein, as shown by a smear detected by Coomassie staining (Figure 1B, left) and western blot (Figure 1B, right). In fact, the smear appearance suggests a mixture of bioconjugate products containing variable numbers of Accum moieties per OVA molecule. This is not surprising, as OVA contains 20 lysine residues (Figure 1C), 16 of which are predicted to be accessible for cross-linking (Figure 1D). Since chemical modifications of proteins can affect their physio-chemical properties, we next assessed the overall stability of aOVA by measuring protein unfolding following thermal stress (intrinsic tryptophan fluorescence [ITF] analysis). In this assay, changes in peak shifts or intensities are indicative of unfolding, as peptide residues may become solvent-exposed and undergo changes in orientation. Compared with nOVA, an increased stability to thermal denaturation was conferred by Accum conjugation as shown with 5X, 10X, and 25X Accum to OVA ratios (Figure 1E). The partial reduction in peak intensity observed at 80°C for the 50X aOVA, could be attributed to non-specific binding of excess Accum to charged peptides, consequently facilitating the denaturation of some aOVA bioconjugates. Since endosomal escape is directly proportional to the number of Accum moieties per target molecule, we elected to conduct all subsequent studies using the 50X aOVA.[12]

Uptake of aOVA ruptures endosomal membranes in mature DCs conferring potent CD4 and CD8 T cell activation

To determine if aOVA enhances endosome-to-cytosol escape, a Galectin-3 (Gal3) system was used as a marker of damaged endo-membranes.[13] More specifically, Gal3 exhibits high affinity toward β-galactosidase conjugates, which are normally present on the cell surface, Golgi apparatus, and in the lumens of endocytic compartments.[13] Therefore, when expressed under normal conditions, Gal3 is evenly distributed across the cytoplasm. Conversely, induction of endosomal membrane rupture allows Gal3 to access and bind luminal glycoproteins.[13] We thus transiently transfected the DC2.4 cell line with a construct expressing the Gal3 as a fusion with the enhanced GFP (eGFP-Gal3) to evaluate its distribution pattern. As anticipated, the GFP signal was diffusely distributed throughout the cytosol following treatment of eGFP-Gal3-expressing DC2.4 cells with nOVA (Figure 2A, upper panel). In contrast, pulsing of DC2.4 with aOVA

CellPress
OPEN ACCESS

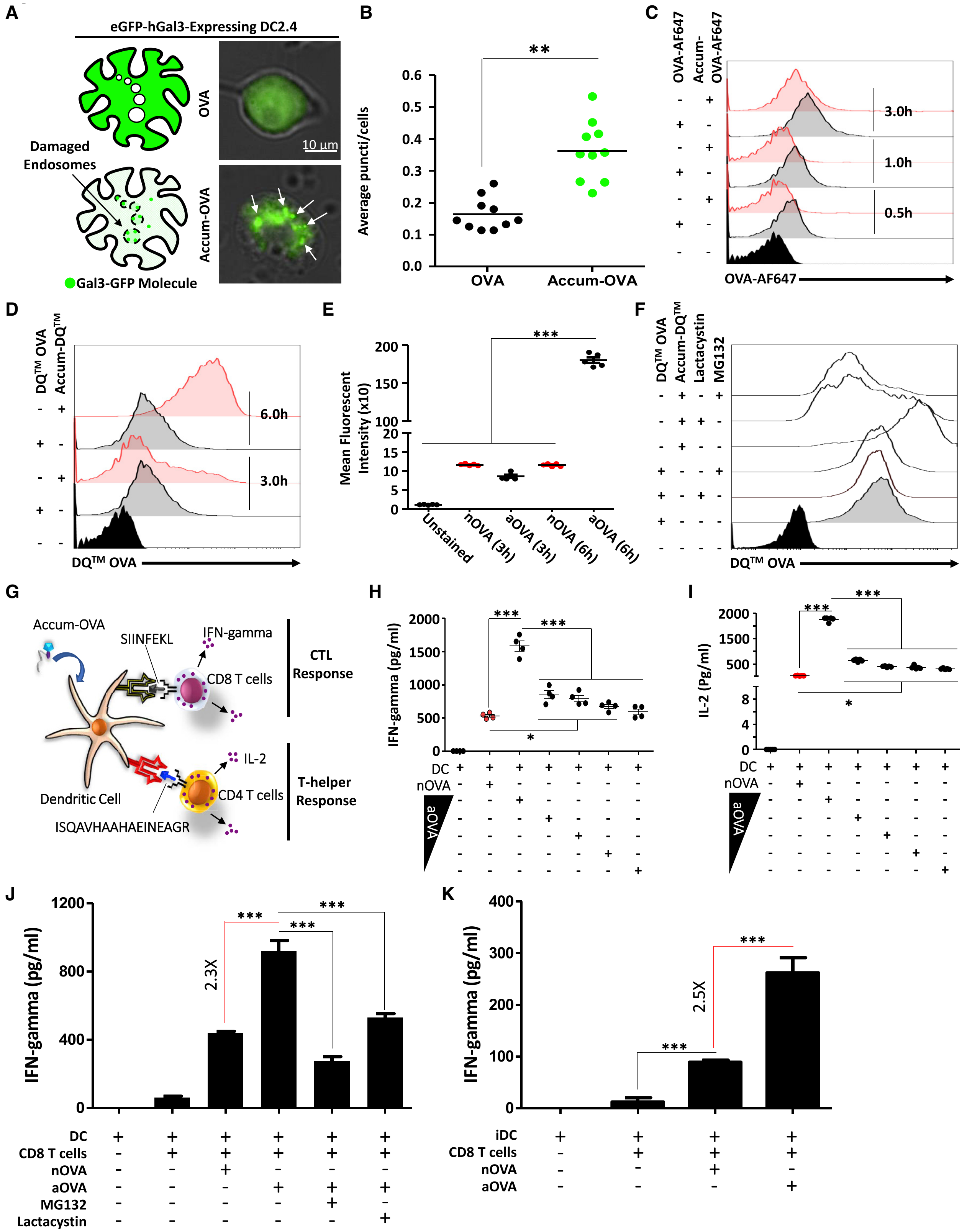

(legend on next page)

induces the appearance of several puncta, clearly indicating signal re-localization to damages endosomes (Figure 2A - lower panel, Figure 2B). To assess whether this enhanced endosomal damage is a cause of high aOVA accumulation in mature DCs, an antigen uptake assay was conducted using fluorescent OVA-AlexaFluor 647 conjugate (AF647). As shown in Figure 2C, mature DCs treated with nOVA-AF647 were always emitting a signal slightly higher than aOVA-AF647, clearly indicating no beneficial effect for Accum on OVA capturing. In light of these observations, we next monitored intracellular processing of captured OVA. For this purpose, the Accum was cross-linked onto OVA-DQ (a protease-sensitive quenched OVA product, which on hydrolysis produces brightly fluorescent products) prior to pulsing *ex vivo* generated primary bone marrow-derived mature DCs (Figure S1). Although no major differences could be depicted for both antigen conditions 3 h post-mature DC pulsing, the signal intensity in mature DCs treated with Accum-linked OVA-DQ was significantly higher 6 h post-pulsing compared with nOVA (Figures 2D and 2E). Interestingly, no differences in signal intensity could be detected between nOVA pulsing at 3 or 6 h, suggesting signal saturation (Figure 2D). To confirm that the enhanced degradation signal observed at 6 h is due to enhanced proteasome degradation as a result of enhanced OVA escape into the cytoplasm, an OVA-DQ experiment was repeated but following mature DC treatment with MG132 or lactacystin (two proteasomal inhibitors). As shown in Figure 2F, inhibitor pre-treatments of mature DCs prior to Accum-OVA-DQ addition significantly lowered the fluorescent signal, clearly indicating that the initially observed degradation was mediated by enhanced proteasomal activity rather than degradation by endosomal proteases. Nevertheless, these observations correlate perfectly with the antigen presentation assays using primary mature DCs co-cultured with OT-I (CD8) or OT-II (CD4) T cells (Figure 2G). More specifically, production of interferon (IFN)-gamma by OT-I (Figure 2H) and interleukin (IL)-2 (Figure 2I) or IFN-gamma (Figure S2) by OT-II were both superior in the aOVA protein groups even at a concentration 10-fold lower than the one used for nOVA (i.e., 0.1 mg/mL versus 1 mg/mL). Addition of MG132 or lactacystin to mature DCs prior to antigen pulsing lowered T cell activation significantly (Figure 2J). This observation reiterates the notion that Accum enhances antigen escape resulting in enhanced proteasomal degradation. To exclude the possibility of Accum acting as a non-specific adjuvant to DCs, an antigen cross-presentation experiment was performed using iDCs with no prior LPS stimulation. Although an increase in T cell activation was also observed in the aOVA group (Figure 2K), the fold change obtained between nOVA and aOVA was similar to the one using mature DCs (2.3 versus 2.5, respectively).

To test whether DCs pulsed with aOVA are consistently superior to nOVA, mature DCs were first pulsed with the antigen formulations then frozen for 30 days prior to their re-testing in an antigen presentation assay (Figure S3A). Although lower in magnitude, a similar T cell activation outcome was observed with OT-I-derived CD8 T cells (Figure S3B). To verify whether the selected Accum structure is optimal, an antigen presentation assay using the SIINFEKL-specific B3Z cell line was conducted to screen various Accum conditions (Figure S4). Although the use of 10-, 25- or 50-fold excess Accum per target protein combined to four or six PEG molecules all lead to similar responses, the obtained signals were higher compared with Accum moieties containing 24 PEG molecules (Figure S4). Collectively, these results indicate that mature DC pulsing with aOVA instills distinctive abilities to cross-present (via MHCI) or present (via MHCII) antigens to responding T cells.

Protein-based prophylactic vaccination controls tumor growth

In light of the enhanced T cell activation observed with aOVA, we next tested whether direct protein injection (prophylactic vaccination) could trigger protective immunity against the OVA-expressing T cell lymphoma line EG.7 (Figure 3A). Indeed, administration of aOVA significantly delayed tumor growth when delivered in the absence of an adjuvant (Figures 3B and 3C), resulting in 30% survival beyond 30 days post-immunization (Figure 3D). On the other hand, vaccination using the two adjuvants AddaSO3 or AddaVax both improved the immune response, with AddaVax triggering superior effects in aOVA-vaccinated mice (Figures 3B–3D). Consistently, serum analysis by ELISA for anti-OVA immunoglobulin G revealed higher titers when adjuvants were co-administered with aOVA (Figure 3E). Although these data clearly demonstrate that adjuvant co-administration with aOVA amplifies ongoing immune responses, aOVA delivery as a stand-alone treatment leads to beneficial anti-cancer

Figure 2. Antigen cross-presentation assay

(A) Representative experiment of Gal3-GFP-expressing DC2.4 cells treated with nOVA (upper image) versus aOVA (lower image). White arrows point to some damaged endosomes.

(B) Puncti quantification per cell in transfected cultures treated with OVA (black circles) or Accum-OVA (green circles). For this experiment, n = 12 fields of view from three different cultures with $^{**}p < 0.01$.

(C) A representative flow cytometry experiment to assess fluorescent OVA-AF647 or Accum-OVA-AF647 uptake by mature DCs.

(D) A representative flow cytometry experiment investigating OVA-DQ versus aOVA-DQ processing by mature DCs.

(E) Quantification of the mean fluorescent intensity of the OVA-DQ/aOVA-DQ signals shown in (D). For this experiment, n = 5/group with $^{***}p < 0.001$.

(F) A representative flow cytometry assessment of OVA-DQ processing following mature DC treatment with proteasome inhibitors.

(G) Schematic diagram showing the set-up of the antigen cross-presentation used to assess OVA-responding OT-I (CD8) and OT-II (CD4) T cells.

(H) IFN-gamma quantification using OT-I-derived CD8 T cells co-cultured with pulsed mature DCs.

(I) IL-2 quantification using OT-II-derived CD4 T cells co-cultured with pulsed mature DCs. For both (H and I), n = 4/group with $^{***}p < 0.001$. The nOVA concentration is 1 mg/mL whereas the aOVA concentrations used were 1, 0.1, 0.05, 0.25, and 0.0125 mg/mL.

(J) IFN-gamma quantification using OT-I-derived CD8 T cells co-cultured with pulsed mature DCs pre-treated with proteasome inhibitors.

(K) Antigen cross-presentation assay using iDCs pulsed with nOVA or aOVA. For (J and K), n = 5/group with $^{***}p < 0.001$. See also Figures S1–S3. All experiments presented in (C–K) were repeated at least three times.

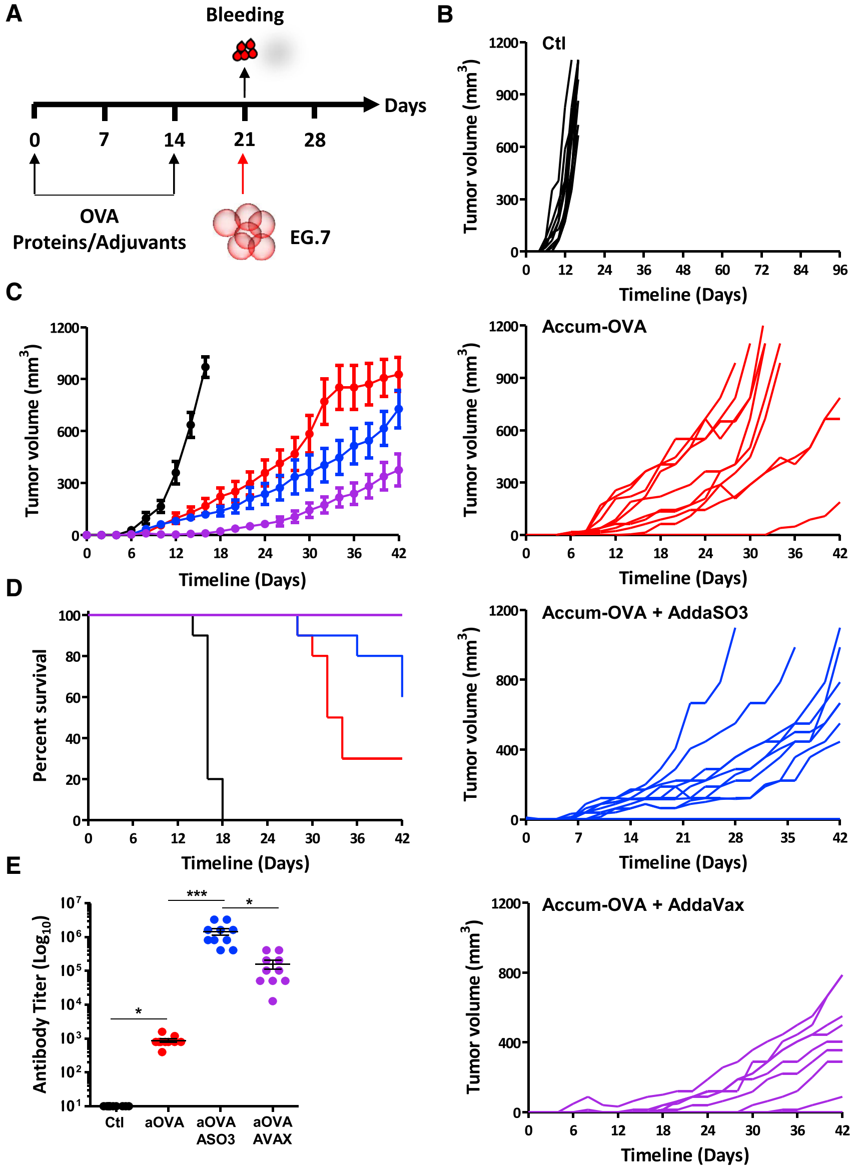

Figure 3. Immunity assessment following direct injection of the OVA protein

(A) Schematic diagram showing the vaccination study design.

(B) Individual tumor measurements in animals immunized using aOVA (1μg - red line), aOVA mixed with AddaSO3^{TM} (blue line) or AddaVax (purple line). Non-immunized mice injected with EG.7 are shown in black.

(C) Average tumor measurements for the experiment shown in (B).

(D) Kaplan Meier survival curve of the experiment displayed in (B and C).

(E) Quantification of antibody titer from the experiment shown in (A–D). For this experiment, n = 10/group with *p < 0.05 and ***p < 0.001. See also Figure S4. All studies presented in (B–E) were repeated two times.

responses, most likely via activation of cellular immunity given the low antibody titer detected in that group.

Prophylactic vaccination using mature DCs pulsed with aOVA leads to long-lasting protection against T cell lymphoma

Monocyte-derived DCs have been extensively used in the design of cellular cancer vaccines.[14,15] Despite their safety profile, the potency of these DC-based vaccines was limited due to various shortcomings, including acidification of their endosomal compartments, which inflicts damage to captured antigens resulting in weakened cross-presentation of immunogenic peptides.[14,15] We thus elected to test the potency of primary mature DCs pulsed with the Accum formulation in the context of prophylactic settings (Figure 4A). Vaccination of naive competent C57BL/6 mice using a low mature DC dose (10^5 cells) pulsed with aOVA triggered complete and long-lasting protection in all vaccinated animals (10 of 10) despite three tumor challenges using ascending EG.7 lymphoma doses at days 21, 42, and 63 post-immunization (Figures 4B, 4C, and S5A). To understand the basis of this potent protection, various immune parameters were assessed. First, immunization using aOVA-pulsed mature DCs triggered a high antibody titer (~1:24,000) compared with the group receiving nOVA-treated mature DCs (1:960 - Figure S5B). Second, the level of CD4 effector ($CD44^{hi}CD62L^{lo}$) and CD8 central ($CD44^{hi}CD62L^{hi}$) and effector memory T cells was substantially higher in the aOVA-DC group (Figure 4D). Finally, Luminex analysis of cytokines/chemokines derived from *in vitro* re-stimulated T cells show elevated levels of IFN-gamma in the aOVA group compared with nOVA-injected mice (Figure 4E). Similar data were also observed for macrophage-inflammatory protein (MIP)-1β and MIP-2, two strong chemoattractants for monocytes/macrophages, natural killer (NK) cells, and neutrophils, as well as IL-6 and IL-10, two cytokines known to support B cell differentiation and antibody production (Figure 4E). Altogether, the improved immune responses observed in animals vaccinated with aOVA-pulsed mature DCs is consistent with their acquired resistance to multiple EG.7 re-challenges and durable survival benefits.

Therapeutic vaccination using syngeneic and allogeneic aOVA-pulsed mature DCs controls tumor growth

Given the impressive protection induced by prophylactic vaccination, we next assessed the vaccine ability to treat animals with pre-established lymphoma tumors (Figure 5A). Following the appearance of palpable masses (~35–50 mm^3), a dose of 3 × 10^5 syngeneic mature DCs pulsed with nOVA or aOVA was administered subcutaneously at days 4 and 11. Although administration of anti-PD-1 or nOVA-pulsed mature DCs as

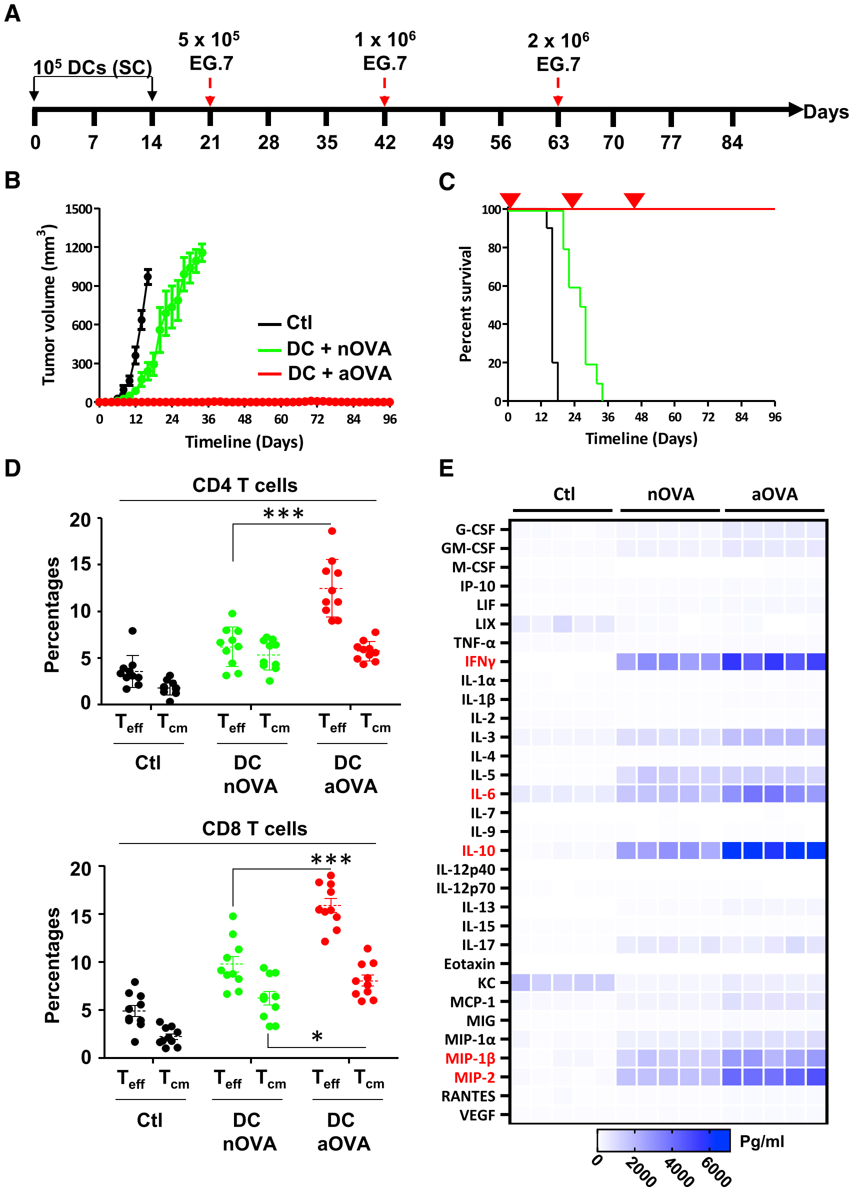

Figure 4. Syngeneic prophylactic vaccination against T cell lymphoma

(A) Schematic representation of the timeline used for prophylactic vaccination using the OVA protein.

(B and C) Assessment of tumor growth volume (B) and survival (C) of animals challenged with the EG.7 tumor following prophylactic vaccination using nOVA-/aOVA-pulsed mature DCs.

(D) Quantification of T_{CM} and T_{eff} CD4 and CD8 T cells derived from mice immunized with nOVA-/aOVA-pulsed mature DCs from vaccinated animals shown in (B and C).

(E) Luminex analysis of cytokine/chemokine production in response to *in vitro* re-stimulation of T cell isolated from vaccinated animals shown in (B and C). Cytokines/chemokines with the highest fold change are depicted in red. For (B–D), n = 10/group with ***p < 0.001. For (E), n = 5/group. See also Figure S5. All studies presented in (B–D) were repeated two times.

single-arm treatments elicited minimal delays in tumor growth, combination of nOVA-DC with anti-PD-1 led to a trivial effect, as it was comparable to the aOVA-DC group (Figures 5B and S6). Conversely, anti-PD-1 co-administration with aOVA-DC cured 20% of animals with established tumors while triggering a partial response (PR) in 30% of vaccinated mice (Figure 5B and S6), with an overall survival rate of 50% (Figure 5C).

Although the use of autologous mature DCs as personalized cancer vaccines remains an appealing approach, their manufacturing process, long timelines, and mass production have greatly limited their translational use.[16] Thus, the development of a one-size-fits-all allogeneic mature DC vaccine represents an interesting alternative. Accordingly, we next asked whether a similar outcome could be reached in the context of allogeneic vaccination. For this purpose, mature DCs derived from BALB/c ($H2^d$) mice were used to vaccinated C57BL/6 mice ($H2^k$) using doses ranging from 3 × 10^3 to 3 × 10^5 cells in combination with the anti-PD-1 antibody. As shown in Figure 5D, the use of allogeneic mature DCs leads to a cure rate comparable to syngeneic vaccination (10% complete response [CR] versus 20%, respectively) along with increased delay in tumor growth (40% PR versus 30%, respectively; Figure 5D). Although lower mature DC doses delayed tumor growth, all vaccinated animals succumbed to death by the end of the experiment (Figures 5D and 5E). These results indicate that immunity conferred by aOVA is superior to nOVA in the context of both syngeneic and allogeneic vaccination.

Allogeneic mature DCs pulsed with tumor lysate confer potent antitumoral immunity

The main goal of cancer immunotherapy is to constrain and/or induce the regression of established tumors. However, the sustained pressure established by effector immune cells toward a defined set of antigens often promotes tumor development through immune-editing and/or immune-escape, consequently resulting in accelerated tumor outgrowth.[17] To broaden the potential use of our vaccine technology, we elected to repeat the vaccination protocol previously established with allogeneic mature DCs using Accum-linked proteins derived from the lysate of the non-OVA-expressing EL4 cells (Figure 6A). Administration of Accum-lysate-pulsed mature DCs to mice with pre-established EL4 tumors led to trivial delays in tumor growth, with all animals dying by day 26 (Figures 6B, 6C, and S7). In contrast, a 30% CR (Figure 6B and S7) with an overall survival of 70% was obtained when the same vaccination strategy was combined with anti-PD-1 (Figure 6C). Interestingly, mature DCs pulsed with standard lysate proteins did not lead to a noticeable therapeutic effect even when co-administered with

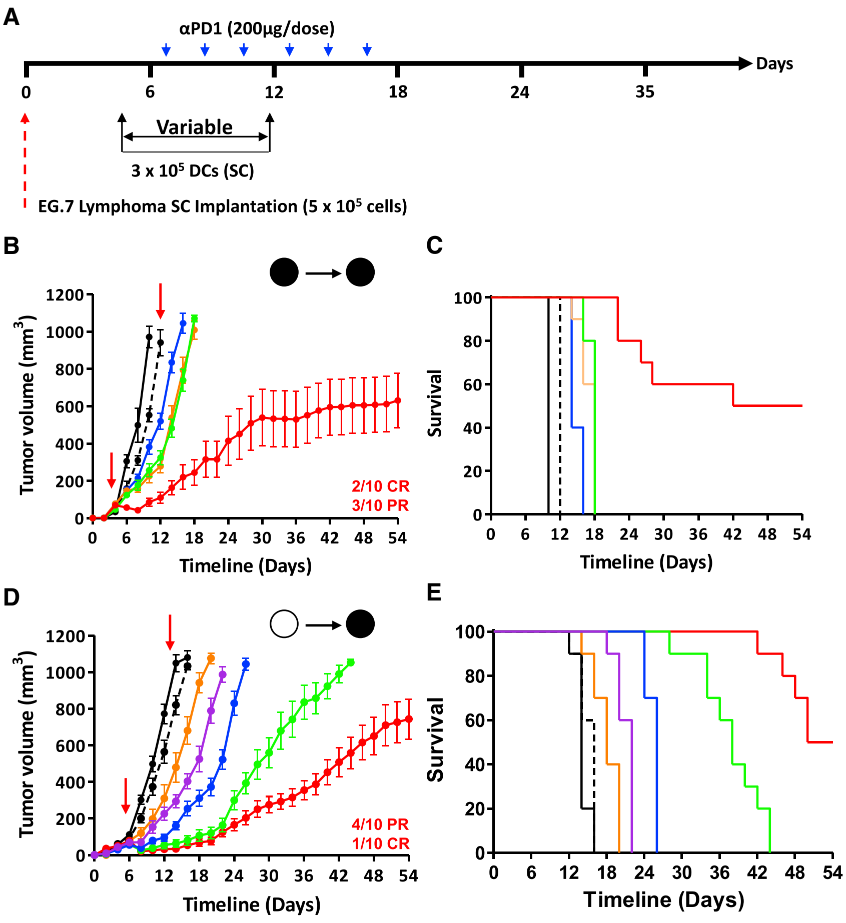

Figure 5. Therapeutic vaccination against T cell lymphoma

(A) Schematic representation of the timeline used for therapeutic vaccination.

(B and C) Assessment of tumor growth volume (B) and survival (C) of animals challenged with the EG.7 tumor following syngeneic therapeutic vaccination using nOVA-/aOVA-pulsed mature DCs. Ctl mice are shown in black, anti-PD-1 in dotted black, DC/nOVA in blue, DC/aOVA in orange, DC/nOVA + anti-PD-1 in green, and DC/aOVA + anti-PD-1 in red.

(D and E) Assessment of tumor growth volume (D) and survival (E) of animals challenged with the EG.7 tumor following allogeneic therapeutic vaccination using nOVA-/aOVA-pulsed mature DCs. Ctl mice are shown in black, anti-PD-1 in dotted black, 300K DC/aOVA in orange, 3K DC/aOVA + anti-PD-1 in purple, 30K DC/aOVA + anti-PD-1 in blue, 100K DC/aOVA + anti-PD-1 in green, and 300K DC/aOVA + anti-PD-1 in red. For all panels, n = 10/group. See also Figure S6.

anti-PD-1. These observations were further supported by analyzing the profile of tumor-infiltrating lymphocytes (TILs) (Figures 6D and S8), which revealed enhanced recruitment of CD8, NK, and CD11c immune effector cells in the Accum-lysate-pulsed DCs/PD-1 (Figure 6E). In sharp contrast, the level of regulatory CD4 T cells (Tregs) was greatly diminished in the same group (Figure 6F), bolstering the idea that combining Accum-lysate-pulsed mature DCs to PD-1 favors inflammation by tipping the balance in favor of CD8 T cells versus suppressive Treg infiltration (Figure 6G). Overall, these findings indicate that "off-the-shelf" allogeneic mature DCs treated with the Accum-lysate formulation can be effectively exploited as universal vaccines to trigger potent anti-tumoral responses.

DISCUSSION

Although most DC subsets exhibit similar antigen capturing capacity, antigen cross-presentation has consistently been superior in specific DC subsets (e.g., $CD8^{+}$DCs in mice or $CD141^{+}XCR1^{+}$ in humans).[8,18] This indicates that antigen delivery to the cytosol is not driven by a common pathway, nor relying on non-specific leakage from endocytic compartments. Instead, the consensus stipulates that antigen routing following endocytosis is a tightly regulated process and depends on the DC subset at play.[18] This dilemma brought forward a series of studies suggesting endosome-to-cytosol antigen migration to be specifically mediated by membrane transporters involving components of the endoplasmic reticulum-associated degradation (ERAD) machinery.[19,20] If we presume that this concept applies to most antigen cross-presentation processes, then how can these channels/pores translocate large antigens to the cytosol? The most plausible explanation lies in the activation of endosomal-resident proteases following acidification of maturing endosomes.[4–8] As such, non-specific antigen degradation is initiated to generate smaller protein fragments adapted to pore sizes. Although this process ensures efficient antigen import to the cytosol, it may inflict collateral damage to immunogenic epitopes within the endocytosed antigen impairing, therefore, T cell activation.[9,20–22] To overcome this barrier, we used the Accum technology to rupture endosomal membranes as a means to avoid protease-mediated damages while promoting antigen escape into the cytosol for efficient proteasomal processing (Graphical abstract). Besides validating our main working hypothesis, our data also allude to the importance of endosome-to-cytosol translocation as a critical and limiting step for antigen cross-presentation and subsequent T cell activation. In addition, our approach provides the impetus to recycle most, if not all, tumor-associated antigen (TAA)-based DC vaccines that did not meet set objectives in past clinical trials. Particular examples include naturally glycosylated forms of MUC1 and/or HER-2/neu, which failed at eliciting T cell responses, as they remained stranded in early endosomes without undergoing proteasomal processing.[23,24]

Besides improving the stability of the antigen (ITF data), Accum improved aOVA processing by monocyte-derived mature DCs, which correlates perfectly with the enhanced IFN-gamma production by responding CD8 T cells. Interestingly, CD4 T cell

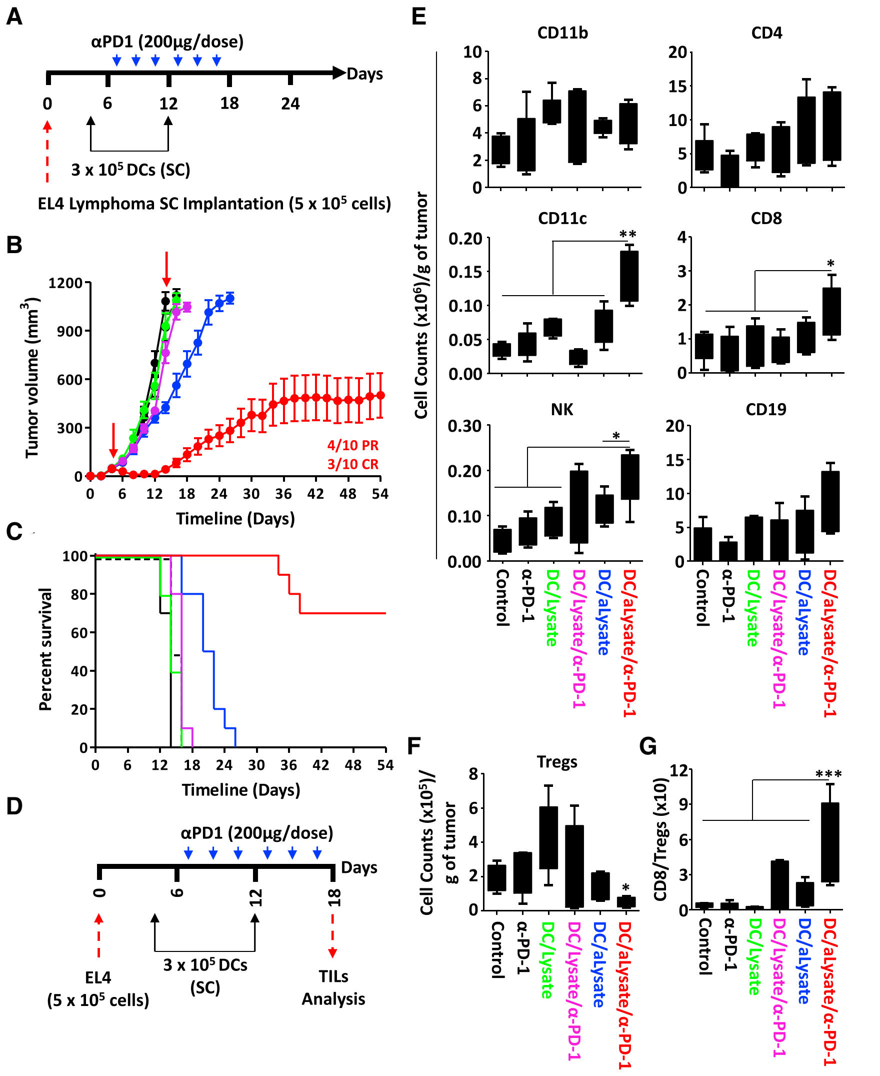

Figure 6. Tumor lysate-based therapeutic vaccination against T cell lymphoma

(A) Schematic representation of the timeline used for allogeneic therapeutic vaccination.

(B and C) Assessment of tumor growth volume (B) and survival (C) of animals challenged with the EL4 tumor following Balb/c-derived allogeneic mature DCs pulsed with EL4 lysate/EL4-Accum-lysate. Ctl mice are shown in black, anti-PD-1 in dotted black, DC/EL4 lysate in green, DC-EL4 Accum-lysate in blue, DC/EL4 lysate + anti-PD-1 in purple, and DC/EL4 Accum-lysate + anti-PD-1 in red.

(D) Schematic representation of the experimental design of the TILs study.

(E) Analysis of various immune cells in tumors derived from all groups shown in (B and C).

(F) Absolute number of Tregs/gram of tumor from all groups shown in (B and C).

(G) Assessment of the CD8/Treg ratio in the tumors depicted in (B and C). For (B and C), n = 10/group. For (E–G), n = 5/group with $^{*}p < 0.05$, $^{**}p < 0.01$, and $^{***}p < 0.001$. See also Figures S7 and S8.

activation was also improved following their co-culture with aOVA-pulsed mature DCs. Although beyond the scope of this study, a possible hypothesis for this improved MHCII-mediated antigen presentation is autophagy induction following damages inflicted to endosomal structures.[20,25–30] Although different from starvation-induced autophagy, this process is selective and involves specific sequestration of cellular components via various mechanisms aimed at repairing, removing, or recycling damaged endosomes.[20,25–28,31] Such mechanisms preserve cellular homeostasis and integrity from the toxicity of released endosomal cargo.[31] We can thus stipulate that endosomal damages triggered by aOVA may cause a specific type of autophagy consequently promoting MHCII antigen presentation.[29,30]

Although DCs have shown promising effects in several preclinical models, the generation of more efficient human or murine cross-presenting DCs at a scale amenable to therapeutic applications while maintaining the desired phenotype and function remains a major deterring factor for translational studies.[32] We thus accounted for this limiting factor by testing (1) the potency of the technology on the most commonly used monocyte-derived mature DCs (often used in the clinic), and (2) low cellular doses. Indeed, prophylactic vaccination using aOVA-pulsed mature DCs elicited potent memory responses, which is in line with the observed complete protection even after three subsequent challenges using ascending cancer cell doses. Therapeutic vaccination, on the other hand, synergized with anti-PD-1 in treating animals with pre-established lymphomas in both syngeneic and allogeneic settings. Besides improving anti-tumoral immunity through enhanced recruitment of effector NK^{+} and $CD8^{+}$ lymphocytes at the expense of regulatory T cells, allogeneic vaccination improved recruitment of endogenous $CD11c^{+}$ cells. This observation is most likely owing to the induction of a pro-inflammatory tumor microenvironment driven by the "adjuvant" effect mediated by allogeneic mature DC administration.[33] As such, inflammation-related damages may lead to epitope spreading and uptake by endogenous DCs further amplifying the anti-tumoral response. These observations highlight the versatility of combining Accum-linked antigens with various checkpoint blockers and covey a major advantage in applying this technology to tumor lysate, thus allowing the development of personalized cancer vaccines without prior identification of specific epitopes or neoantigens.

Limitations of the study

Antigen cross-presentation is a complex field of research due to the various non-mutually exclusive antigen cross-presentation pathways described so far. Whether mediated by phagosome-to-cytosol, recycling endosomes, the vacuolar-related pathway, endoplasmic reticulum-related (including recruitment of specific machinery to endosome), or autophagy, it is generally difficult to

target one specific pathway over another.[34] We developed, tested, and validated the use of a distinctive molecular tool capable of eliciting endosomal damages, which in turn, results in proper antigen release in the cytosol where it becomes accessible to the proteasome. However, we observed beneficial effects on MHCII antigen presentation indicating the potential involvement of more than one pathway in response to Accum-linked antigen use. Nevertheless, the cross-presentation potential of monocyte-derived mature DCs was enhanced without the need of complex pro-inflammatory stimuli or laborious manufacturing to generate naturally scarce cross-presenting DC subsets.[35,36] Additional studies are however needed to test other TAAs prior to deriving further conclusions on Accum use as an enhancer for DC vaccine preparation. Notwithstanding, our study provides an immunotherapeutic proof-of-concept strategy, which could re-define the use of novel or previously developed DC cancer vaccines.

STAR★METHODS

Detailed methods are provided in the online version of this paper and include the following:

- KEY RESOURCES TABLE
- RESOURCE AVAILABILITY
  - Lead contact
  - Materials availability
  - Data and code availability
- EXPERIMENTAL MODEL AND SUBJECT DETAILS
  - Mice strains
  - Cell lines
  - Generation of bone marrow-derived DCs
  - Immunization and tumor challenge studies
- METHOD DETAILS
  - DC2.4 transfection and assessment of damaged endosomes by microscopy
  - Accum synthesis and generation of the Accum-antigen formulations
  - Assessment of Intrinsic Tryptophan fluorescence (ITF)
  - Phenotypic assessment of generated mature DCs by flow cytometry
  - Monitoring antigen uptake and processing
  - Antigen cross-presentation assay
  - Cancer cell lysate preparation
  - Quantification of antibody titer by ELISA
  - Analysis of tumor-infiltrating immune cells
  - Modeling accessible lysine in protein antigen
- QUANTIFICATION AND STATISTICAL ANALYSIS
  - Statistical analysis

SUPPLEMENTAL INFORMATION

Supplemental information can be found online at https://doi.org/10.1016/j.xcrm.2022.100534.

ACKNOWLEDGMENTS

We thank Isabelle Caron and Myriam Métivier-Bélisle from the Institute for Research in Immunology and Cancer animal facility for their kind help with some of the *in vivo* experiments. Studies were funded by the Research and Development Branch of Defence Therapeutics Inc., Vancouver, BC, Canada, and by a research contract to the Laboratory of Dr. M. Rafei (RB002793).

AUTHOR CONTRIBUTIONS

J.P.B., N.S., and S.B. conducted most of the *in vitro* and *in vivo* assays. J.A., M.B., and T.C. were involved in some *in vitro* and *in vivo* experiments (TILs and microscopy respectively). S.T., R.L., and S.P. contributed to data analysis. M.R. conceived and supervised the project, analyzed data, and wrote the first draft of the manuscript. All authors contributed to manuscript editing.

DECLARATION OF INTERESTS

S.B. and S.P. are current employees of Defence Therapeutics Inc. and declare competing financial interest.

Received: June 11, 2021
Revised: December 17, 2021
Accepted: January 27, 2022
Published: February 25, 2022

REFERENCES

1. Bevan, M.J. (2010). Cross-priming for a secondary cytotoxic response to minor H antigens with H-2 congenic cells which do not cross-react in the cytotoxic assay. J. Immunol. *185*, 1361–1366.
2. Carbone, F.R., and Heath, W.R. (2010). Cross-priming: its beginnings. J. Immunol. *185*, 1353–1354.
3. Trombetta, E.S., Ebersold, M., Garrett, W., Pypaert, M., and Mellman, I. (2003). Activation of lysosomal function during dendritic cell maturation. Science *299*, 1400–1403.
4. Jancic, C., Savina, A., Wasmeier, C., Tolmachova, T., El-Benna, J., Dang, P.M., Pascolo, S., Gougerot-Pocidalo, M.A., Raposo, G., Seabra, M.C., et al. (2007). Rab27a regulates phagosomal pH and NADPH oxidase recruitment to dendritic cell phagosomes. Nat. Cell Biol. *9*, 367–378.
5. Mantegazza, A.R., Savina, A., Vermeulen, M., Pérez, L., Geffner, J., Hermine, O., Rosenzweig, S.D., Faure, F., and Amigorena, S. (2008). NADPH oxidase controls phagosomal pH and antigen cross-presentation in human dendritic cells. Blood *112*, 4712–4722.
6. Rybicka, J.M., Balce, D.R., Chaudhuri, S., Allan, E.R., and Yates, R.M. (2012). Phagosomal proteolysis in dendritic cells is modulated by NADPH oxidase in a pH-independent manner. EMBO J *31*, 932–944.
7. Savina, A., Jancic, C., Hugues, S., Guermonprez, P., Vargas, P., Moura, I.C., Lennon-Duménil, A.M., Seabra, M.C., Raposo, G., and Amigorena, S. (2006). NOX2 controls phagosomal pH to regulate antigen processing during crosspresentation by dendritic cells. Cell *126*, 205–218.
8. Savina, A., Peres, A., Cebrian, I., Carmo, N., Moita, C., Hacohen, N., Moita, L.F., and Amigorena, S. (2009). The small GTPase Rac2 controls phagosomal alkalinization and antigen crosspresentation selectively in CD8(+) dendritic cells. Immunity *30*, 544–555.
9. Dingjan, I., Verboogen, D.R., Paardekooper, L.M., Revelo, N.H., Sittig, S.P., Visser, L.J., Mollard, G.F., Henriet, S.S., Figdor, C.G., Ter Beest, M., et al. (2016). Lipid peroxidation causes endosomal antigen release for cross-presentation. Sci. Rep. *6*, 22064.
10. Silk, K.M., Silk, J.D., Ichiryu, N., Davies, T.J., Nolan, K.F., Leishman, A.J., Carpenter, L., Watt, S.M., Cerundolo, V., and Fairchild, P.J. (2012). Cross-presentation of tumour antigens by human induced pluripotent stem cell-derived CD141(+)XCR1+ dendritic cells. Gene Ther. *19*, 1035–1040.
11. Sachamitr, P., Hackett, S., and Fairchild, P.J. (2014). Induced pluripotent stem cells: challenges and opportunities for cancer immunotherapy. Front. Immunol. *5*, 176.
12. Beaudoin, S., Rondeau, A., Martel, O., Bonin, M.A., van Lier, J.E., and Leyton, J.V. (2016). ChAcNLS, a novel modification to antibody-conjugates

permitting target cell-specific endosomal escape, localization to the nucleus, and enhanced total intracellular accumulation. Mol. Pharm. *13*, 1915–1926.

13. Paz, I., Sachse, M., Dupont, N., Mounier, J., Cederfur, C., Enninga, J., Leffler, H., Poirier, F., Prevost, M.C., Lafont, F., et al. (2010). Galectin-3, a marker for vacuole lysis by invasive pathogens. Cell Microbiol. *12*, 530–544.
14. Anguille, S., Smits, E.L., Lion, E., van Tendeloo, V.F., and Berneman, Z.N. (2014). Clinical use of dendritic cells for cancer therapy. Lancet Oncol. *15*, e257–e267.
15. Figdor, C.G., de Vries, I.J., Lesterhuis, W.J., and Melief, C.J. (2004). Dendritic cell immunotherapy: mapping the way. Nat. Med. *10*, 475–480.
16. Menez-Jamet, J., Gallou, C., Rougeot, A., and Kosmatopoulos, K. (2016). Optimized tumor cryptic peptides: the basis for universal neo-antigen-like tumor vaccines. Ann. Transl. Med. *4*, 266.
17. Schreiber, R.D., Old, L.J., and Smyth, M.J. (2011). Cancer immunoediting: integrating immunity's roles in cancer suppression and promotion. Science *331*, 1565–1570.
18. Schnorrer, P., Behrens, G.M., Wilson, N.S., Pooley, J.L., Smith, C.M., El-Sukkari, D., Davey, G., Kupresanin, F., Li, M., Maraskovsky, E., et al. (2006). The dominant role of CD8+ dendritic cells in cross-presentation is not dictated by antigen capture. Proc. Natl. Acad. Sci. U S A *103*, 10729–10734.
19. Kozik, P., Gros, M., Itzhak, D.N., Joannas, L., Heurtebise-Chrétien, S., Krawczyk, P.A., Rodríguez-Silvestre, P., Alloatti, A., Magalhaes, J.G., Del Nery, E., et al. (2020). Small molecule enhancers of endosome-to-cytosol import augment anti-tumor immunity. Cell Rep. *32*, 107905.
20. Zehner, M., Marschall, A.L., Bos, E., Schloetel, J.G., Kreer, C., Fehrenschild, D., Limmer, A., Ossendorp, F., Lang, T., Koster, A.J., et al. (2015). The translocon protein Sec61 mediates antigen transport from endosomes in the cytosol for cross-presentation to CD8(+) T cells. Immunity *42*, 850–863.
21. Delamarre, L., Pack, M., Chang, H., Mellman, I., and Trombetta, E.S. (2005). Differential lysosomal proteolysis in antigen-presenting cells determines antigen fate. Science *307*, 1630–1634.
22. Rodriguez, A., Regnault, A., Kleijmeer, M., Ricciardi-Castagnoli, P., and Amigorena, S. (1999). Selective transport of internalized antigens to the cytosol for MHC class I presentation in dendritic cells. Nat. Cell Biol. *1*, 362–368.
23. Hiltbold, E.M., Alter, M.D., Ciborowski, P., and Finn, O.J. (1999). Presentation of MUC1 tumor antigen by class I MHC and CTL function correlate with the glycosylation state of the protein taken up by dendritic cells. Cell Immunol. *194*, 143–149.
24. Hiltbold, E.M., Vlad, A.M., Ciborowski, P., Watkins, S.C., and Finn, O.J. (2000). The mechanism of unresponsiveness to circulating tumor antigen MUC1 is a block in intracellular sorting and processing by dendritic cells. J. Immunol. *165*, 3730–3741.
25. Chauhan, S., Kumar, S., Jain, A., Ponpuak, M., Mudd, M.H., Kimura, T., Choi, S.W., Peters, R., Mandell, M., Bruun, J.A., et al. (2016). TRIMs and galectins globally cooperate and TRIM16 and galectin-3 Co-direct autophagy in endomembrane damage homeostasis. Dev. Cell *39*, 13–27.
26. Fujita, N., Morita, E., Itoh, T., Tanaka, A., Nakaoka, M., Osada, Y., Umemoto, T., Saitoh, T., Nakatogawa, H., Kobayashi, S., et al. (2013). Recruitment of the autophagic machinery to endosomes during infection is mediated by ubiquitin. J. Cell Biol. *203*, 115–128.
27. Jia, J., Claude-Taupin, A., Gu, Y., Choi, S.W., Peters, R., Bissa, B., Mudd, M.H., Allers, L., Pallikkuth, S., Lidke, K.A., et al. (2020). Galectin-3 coordinates a cellular system for lysosomal repair and removal. Dev. Cell *52*, 69–87.e8.
28. Skowyra, M.L., Schlesinger, P.H., Naismith, T.V., and Hanson, P.I. (2018). Triggered recruitment of ESCRT machinery promotes endolysosomal repair. Science *360*, eaar5078.
29. Crotzer, V.L., and Blum, J.S. (2009). Autophagy and its role in MHC-mediated antigen presentation. J. Immunol. *182*, 3335–3341.
30. Dengjel, J., Schoor, O., Fischer, R., Reich, M., Kraus, M., Müller, M., Kreymborg, K., Altenberend, F., Brandenburg, J., Kalbacher, H., et al. (2005). Autophagy promotes MHC class II presentation of peptides from intracellular source proteins. Proc. Natl. Acad. Sci. U S A *102*, 7922–7927.
31. Anding, A.L., and Baehrecke, E.H. (2017). Cleaning house: selective autophagy of organelles. Dev. Cell *41*, 10–22.
32. Perez, C.R., and De Palma, M. (2019). Engineering dendritic cell vaccines to improve cancer immunotherapy. Nat. Commun. *10*, 5408.
33. Fotaki, G., Jin, C., Ramachandran, M., Kerzeli, I.K., Karlsson-Parra, A., Yu, D., et al. (2018). Pro-inflammatory allogeneic DCs promote activation of bystander immune cells and thereby license antigen-specific T-cell responses. Oncoimmunology *7*, e1395126.
34. Blander, J.M. (2018). Regulation of the cell biology of antigen cross-presentation. Annu. Rev. Immunol. *36*, 717–753.
35. Fotaki, G., Jin, C., Kerzeli, I.K., Ramachandran, M., Martikainen, M.M., Karlsson-Parra, A., Yu, D., and Essand, M. (2018). Cancer vaccine based on a combination of an infection-enhanced adenoviral vector and pro-inflammatory allogeneic DCs leads to sustained antigen-specific immune responses in three melanoma models. Oncoimmunology *7*, e1397250.
36. Fröbom, R., Berglund, E., Berglund, D., Nilsson, I.L., Åhlén, J., von Sivers, K., Linder-Stragliotto, C., Suenaert, P., Karlsson-Parra, A., and Bränström, R. (2020). Phase I trial evaluating safety and efficacy of intratumorally administered inflammatory allogeneic dendritic cells (ilixadencel) in advanced gastrointestinal stromal tumors. Cancer Immunol. Immunother. *69*, 2393–2401.

STAR★METHODS

KEY RESOURCES TABLE

REAGENT or RESOURCE	SOURCE	IDENTIFIER
**Antibodies**		
CD3 antibody	BD Biosciences	Cat#: 555275, RRID:AB_395699
CD11c antibody	BD Biosciences	Cat#: 550261, RRID:AB_398460
CD19 antibody	BD Biosciences	Cat#: 557399, RRID:AB_396682
Mouse Anti-NK-1.1	BD Biosciences	Cat#: 551114, RRID:AB_394052
CD62L antibody	BD Biosciences	Cat#: 560513, RRID:AB_10611578
I-A/I-E antibody	BD Biosciences	Cat#:562367, RRID:AB_11152078
Rat Anti- mouse CD44 antibody	BD Biosciences	Cat#: 559250, RRID:AB_398661
Mouse H-2K[b] antibody	BD Biosciences	Cat#: 562942, RRID:AB_2737908
Anti-Mouse CD80 antibody	BD Biosciences	Cat#: 553769, RRID:AB_395039
PE Rat Anti-Mouse CD86 antibody	BD Biosciences	Cat#: 553692, RRID:AB_394994
Goat Anti-Mouse IgG HRP antibody	R&D systems	Cat#: HAF007, RRID:AB_357234
*InVivo*MAb anti-mouse PD-1 (CD279)	Bio X cell	Cat#: BE0146, RRID:AB_10949053
**Chemicals, peptides, and recombinant proteins**		
Clodronate liposomes and control liposomes	Liposoma	CSKU: CP-005-005
Recombinant Murine GM-CSF	PeproTech	Cat#: 315-03
Albumin from chicken egg white	Sigma-Aldrich	Cat#: A5503
Lipopolysaccharides from *Escherichia coli* O111:B4	Sigma-Aldrich	Cat#: L2630-10MG
Chlorophenicol red-β-D-galactopyranoside (CPRG)	Sigma-Aldrich	REF#: 10884308001
DNase type IV	Sigma-Aldrich	Cat#: D5025
Collagenase IV	Sigma-Aldrich	Cat#: C4-BIOC
Collagenase D	Sigma-Aldrich	Cat#: COLLD-RO
Cholic acid	Sigma-Aldrich	Cat#: C-1254
Tetramethylbenzidine Liquid Substrate System for ELISA	Sigma-Aldrich	Cat#: T0440-1L
MG132	Tocris Bioscience	Cat#: 1748/5
Lactacystin	Tocris Bioscience	Cat#: 2267
DQ^{TM} ovalbumin	ThermoFisher Scientific	Cat#: D12053
Ovalbumin, Alexa Fluor^{TM} 647 Conjugate	ThermoFisher Scientific	Cat#: O34784
AddaS0$_3^{TM}$ (AS03 -like squalene-based adjuvant)	InvivoGen	Cat#: vac-as03-10
AddaVax^{TM} (Squalene-oil-in-water)	InvivoGen	Cat#: vac-adx-10
SIINFEKL peptide	GenScript	Lot#: U4778GB210-1/PE6339
Bovine Serum Albumin (BSA)	Bio Basic Canada Inc.	CAS#: 9048-46-8
β-Mercaptoethanol	Gibco	Cat#: 21985023
Sodium Pyruvate	Multicell	Cat#: 600-110-EL
Fetal Bovin Serum (FBS)	Multicell	Cat#: 090150
Phosphate Buffered Saline (PBS)	Multicell	Cat#: 311-010-CL
RPMI 1640	Multicell	Cat#: 350-000-CL
Penicillin/Streptomycin	Multicell	Cat#: 450-201-EL
L-glutamine	Multicell	Cat#: 609-065-EL
HEPES	Multicell	Cat#: 330-050-EL
MEM essential amino acid	Multicell	Cat#: 321-011-EL
DMEM	Multicell	Cat#: 319-005-CL
Bradford reagent	Bio-RAD	Cat#: 500-0006
Red blood cell lysis buffer	BioLegend	Cat#: 420301
Stop solution	R&D systems	Cat#: DY008

(*Continued on next page*)

Continued

REAGENT or RESOURCE	SOURCE	IDENTIFIER
Coomasie Blue R-250	aMReSCO	Cat#: M128-25G
Tween20™	aMReSCO	Cat#: 0777-1L
Skim milk	Selection	Lot#: 231-03
Critical commercial assays		
RNeasy Mini Kit (50)	Qiagen	Cat#: 74104
Mouse IFN-gamma DuoSet ELISA	R&D systems	Cat#: DY485-05
Mouse IL-2 DuoSet ELISA	R&D systems	Cat#: DY402-05
EasySep™ Mouse CD8a Positive Selection Kit II	Stemcell Technologies	Cat#:18953 and 18753
EasySep™ FITC for CD4 isolation	Stemcell Technologies	Cat#: 18558
Materials		
G26 needle	Terumo	Cat#: NN2613R
0.45uM filter	UltiDent Scientific	Cat#: 229751
70uM cell strainer	ThermoFisher Scientific	Cat#: 22-363-548
Nunc MaxiSorpTM plates	ThermoFisher Scientific	Lot#: 1311523
96 wells culture plate	Sarstedt AG&CO KG	REF#: 82.1582.100
24 wells plate	Sarstedt AG&CO KG	REF#: 83.3922.300
Experimental models: Cell lines		
Mouse: E.G7-OVA [derivative of EL4]	ATCC	ATCC Cat# CRL-2113, RRID: CVCL_3505
Mouse: EL4	ATCC	Cat# TIB-39, RRID: CVCL0255
Mouse: B3Z	Gift from Dr. Etienne Gagnon	N/A
Mouse: DC2.4	Sigma	SCC142
Experimental models: Organisms/strains		
Mouse: BALB/cAnCrl	Charles River	Strain code: 028
Mouse: C57BL/6NCrl	Charles River	Strain code: 027
Mouse: OT-I (C57BL/6-Tg(TcraTcrb)1100Mjb/J)	The Jackson Laboratory	Strain #003831
Mouse: OT-II (B6.Cg-Tg(TcraTcrb)425Cbn/J)	The Jackson Laboratory	Strain #004194
Recombinant DNA		
eGFP-hGal3	Addgene	Plasmid #73080
Software and algorithms		
FlowJo v10	FlowJo™	https://www.flowjo.com/solutions/flowjo/downloads
Prism-GraphPad	GraphPad Software	https://www.graphpad.com/scientific-software/prism/
MATLAB	MathWorks	https://www.mathworks.com/products/matlab.html
SWISS-MODEL	SWISS-MODEL	https://swissmodel.expasy.org/
RCSP PDB	RCSP PDB	https://www.rcsb.org/3d-view/

RESOURCE AVAILABILITY

Lead contact

Further information and requests for resources and reagents should be directed to and will be fulfilled by the Lead Contact, Moutih Rafei (moutih.rafei.1@umontreal.ca).

Materials availability

The eGFP-hGal3 mammalian expression vector was kindly provided by Dr. Tamotsu Yoshimori (Osaka University, Osaka, Japan).

Data and code availability

- Original microscopy data reported in this paper will be shared by the lead contact upon request.
- This paper does not report original code. Antigen 3D structure modeling and accessible amino acids identification were done using previously available RCSB PDB and Swiss-Model Expasy free access software.
- Any additional information required to reanalyze the data reported in this paper is available from the lead contact upon request.

CellPress
OPEN ACCESS

EXPERIMENTAL MODEL AND SUBJECT DETAILS

Mice strains

For all experiments, Six- to eight-week-old female Balb/c mice and female C57BL/6 mice of similar age were purchased from Charles River (Montreal, QC, Canada), whereas OT-1 (B6.129P2-H2-K1^{tm1Bpe} H2-D1^{tm1Bpe}/DcrJ) and OT-II (B6. Cg-Tg (TcraTcrb)425Cbn/J) mice were purchased from Jackson Laboratories (Bar Harbor, ME, USA). Littermate mice were interbred and housed and maintained in accordance with the guidelines approved by the Animal Care Committee of Université de Montréal in a pathogen-free environment at the animal facility of the Institute for Research in Immunology and Cancer (IRIC). Animal protocols were approved by the Animal Care Committee of Université de Montréal.

Cell lines

EL4, EG.7 used in this study were obtained from ATCC. DC2.4 were purchased from Sigma and B3Z cells were a generous gift from Dr. Etienne Gagnon (Université de Montréal, Montreal, QC, Canada). EL4, B3Z, and DC2.4 cells were maintained in Roswell Park Memorial Institute (RPMI) 1640 Medium supplemented with 10% fetal bovine serum (FBS). E.G7 cells were cultured RPMI 1460 supplemented with 2 g/L Glucose, 10% FBS, 50 U/mL Penicillin-Streptomycin, 2 mM L-glutamine, 10 mM HEPES, 1 mM Sodium Pyruvate, and 0.5 mM β-Mercaptoethanol, and kept under selection using 80 mg/mL of G418. All cells were maintained at 37 °C in a 5% CO2 incubator. All cell culture media and reagents were purchased from Wisent Bioproducts (St-Bruno, QC, Canada).

Generation of bone marrow-derived DCs

Mouse primary mature DCs were generated *ex vivo* by flushing the whole marrow from female C57BL/6 or Balb/c mice femurs using RPMI 1640 supplemented with 10% fetal bovine serum (FBS), 50 U/mL Penicillin-Streptomycin, 2 mM L-glutamine, 10 mM HEPES, 1% MEM Non-essential Amino Acids, 1 mM Sodium Pyruvate, 0.5 mM β-Mercaptoethanol. Following red blood cell lysis, nucleated cells were cultured in media supplemented with 50 ng/mL murine recombinant granulocyte macrophage-colony stimulating factor (GM-CSF). The media was replaced on days 2, 4, 6 and 8 with fresh media containing GM-CSF. To stimulate DC maturation, the media was replaced on day 9 to include recombinant murine GM-CSF (50 ng/mL) and LPS from *Escherichia coli* O111 (1 ng/mL). The phenotype of mature DCs was assessed by flow cytometry for the expression of CD3, CD11c, CD19, CD80, CD86, NK1.1, H2-K^b and I-A^b. Immature (i)DCs were generated following the same protocol up to day 8 then collected without stimulation using LPS.

Immunization and tumor challenge studies

For prophylactic vaccination, female C57BL/6 mice (n = 10/group) were subcutaneously (SC)-injected at days 0 and 14 with nOVA or aOVA (1 μg/dose) or 10^4 mature DCs pulsed with the OVA formulations (0.1 mg/mL) or tumor lysates (0.1 mg/mL). For protein-based immunization studies using adjuvants, 1 μg of nOVA or aOVA was mixed in a 1:1 volume ratio with the AddaVax™ or AddaSO3™ adjuvants. A total volume of 100 μL mix was then injected SC in immunocompetent naïve C57BL/6 mice using the same schedule for all prophylactic vaccinations. Two weeks following the second vaccination, the mice were SC challenged with 5×10^5 EG.7 or EL4 cells and tumor growth was assessed over time using a digital caliper. To evaluate antigen-specific CD8 T-cell activation, splenocytes isolated from immunized mice were first stimulated *in vitro* with 1 μg/mL nOVA then the supernatant collected three days later to assess cytokine/chemokine production by Luminex (Eve Technologies, Calgary, Canada).

For therapeutic vaccination, female C57BL/6 mice (n = 10/group) received a SC injection of 5×10^5 EL4 or EG.7 cells at day 0. Five days later (appearance of palpable tumors ~35–50 mm^3), mice were SC-injected with 3×10^4 nOVA-, aOVA- or tumor lysate-/Accum-lysate-pulsed mature DCs (two injections 1 week apart). Control animals received 5×10^5 tumor cells alone. Treated animals were followed thereafter for tumor growth. For therapeutic vaccination in combination with the immune-checkpoint inhibitors (anti-PD-1), mice received SC-injections of the antibody or its isotype at 200 μg/per dose every 2 days for a total of 6 doses over two weeks. A similar approach was conducted for allogeneic dosing vaccination in Balb/c mice.

METHOD DETAILS

DC2.4 transfection and assessment of damaged endosomes by microscopy

For this assay, 15×10^3 DC2.4 cells were seeded on a sterile cover slide in a 24-well plate. The cells were transfected with the eGFP-hGal3 mammalian expression vector using Polyfect® (Qiagen) following manufacturer instructions. Two days following transfection, 0.1 mg/mL of nOVA or aOVA was added to the cells then incubated for 3 hours at 37°C. The cells were then washed twice to remove excess protein prior to being mounted on a slide using mounting media. The slides were analyzed using a fluorescent Ti2 microscope (Nikon) with a 60X objective. Pictures of four random spots were taken for each culture dish. The cells were identified based on their GFP fluorescence. For each field of view, the total number of puncti and total number of cells were manually counted, and the ratio puncti/cell calculated.

Accum synthesis and generation of the Accum-antigen formulations

Accum was synthesized as previously described.[12] All chemicals, resin and solvents were used as received from suppliers. Fmoc-protected amino acids, diisopropylethylamine (DIPEA), 2-(1H-7-Azabenzotriazol-1-yl)-1,1,3,3-tetramethyl uronium

hexafluorophosphate methanaminium (HATU) and trifluoroacetic acid (TFA) were purchased from Chem-impex international (Wood Dale, IL). The rink amide resin was obtained from Rapp Polymere (Tübingen, Germany). ChAc, triisopropylsilane (TIPS) and ethanedithiol (EDT), were obtained from Sigma-Aldrich (St-Louis, MO). Dimethylformamide (DMF), isopropanol (IPA) and dichloromethane (DCM) were purchased from VWR (Québec, Canada). Piperidine was obtained from A&C Chemicals (Québec, Canada). UPLC-MS analyses were performed with a Waters (Milford, MA) AQUITY H-class – SQD2 mass detector and PDA eλ UV-visible detector on a BEH, C18, 1.7 μm, 2.1 × 50 mm. Purifications were performed on a Waters preparative UPLC system consisting of injector 2707, pump 2535, and detector 2489, with an ACE C18 column 250 × 21.2 mm, 5 μm (Canadian Life Science, Ontario, Canada). For analytical UPLC, water and acetonitrile with 0.1% formic acid were used. For preparative UPLC, water plus 0.1% TFA, and pure acetonitrile were used. Peptide syntheses were performed on Tribute UV-IR automated peptide synthesizer from Protein Technologies (Tucson, AZ) following manufacturer's recommendations. Peptides were synthetized on the solid phase Rink Amide resin (loading 0.22 mmol/g) using an automated Tribute UV-IR Peptide Synthesizer, at 50 μmol scale. Fmoc groups deprotection was achieved using 20% piperidine in DMF using the UV monitoring smart deprotection feature. Couplings were performed using 5 eq of amino acids, activated with HATU and DIPEA (1:2 molar ratio in relation to the amino acid) for 2 minutes with IR heating at 50°C (except for Fmoc-Cys (Trt), 20 minutes at room temperature). The final deprotection was performed manually using 50% piperidine in DMF for 30 minutes and resin were washed using DMF x2, DCM x3, and IPA. The ChAc unit was coupled using 5 eq of the acid, activated with HATU and DIPEA (1:2 molar ratio in relation to the ChAc) for 16 hours and then resin was washed as described above. The peptides were cleaved from their solid support using a mixture of TFA / H2O / TIPS / EDT (92.5 / 2.5 / 2.5 / 2.5) (4 mL for 200 mg of resin) for 3 hours. Crude peptides were precipitated in chilled diethyl ether, centrifuged, and allowed to dry prior to reverse phase preparative UPLC purification. Final peptides were characterized using mass spectroscopy and UPLC.

The OVA, OVA-AF647, OVA-DQ, or cancer cell lysate were solubilized at 1–10 mg/mL in sterile phosphate buffer saline (PBS) with or without other formulation components but free of amine or sulfhydryl group. The SM(PEG)4 cross-linker was added to the reaction for 1 hour using different molar excess ratio (5X, 10X, 25X, 50X). The free SM(PEG)4 cross-linker was discarded by centricon filtration and Sephadex column. Accum was added in the same molar excess ratio and incubated for 1 hour to obtain different amount of Accum moieties linked per antigen. Free unlinked Accum was removed by centricon filtration and Sephadex column. Accum-modified antigens were concentrated in sterile PBS to obtain final concentration 5–10 mg/mL as determined by ultra-violet absorbance.

To evaluate the bioconjugation efficiency, 10 μg of nOVA or aOVA conjugate were loaded under reducing conditions onto a 12% polyacrylamide gel and stained with Coomassie brilliant blue R-250 (Bio-Rad, Mississauga, ON, Canada). The migration distance in the gel relative to the blue dye front (Rf) was measured and the numbers of Accum moieties per OVA molecule were categorized into low, medium, and high Accum loads estimated by reference to a logarithm plot of molecular weight versus 1/Rf for Kaleidoscope prestained standards (Bio-Rad) electrophoresed under identical conditions. In addition, western blot against OVA was performed to confirm the Coomassie results.

Assessment of Intrinsic Tryptophan fluorescence (ITF)

An Applied Photophysics (Leatherhead, Surrey, UK) Chirascan Q100 circular dichroism spectrometer was used for ITF analysis and a VWR digital heatblock (Radnor, PA) was used for dry block temperature incubations. The Chirscan Q100 autosampler rack cooling system was used for all 4°C incubations. Data was analyzed using MATLAB software (Natick, MA). Briefly, the samples were removed from storage at −20°C and allowed to equilibrate to room temperature, before their dilution to 0.8 mg/mL in PBS. Stock concentrations of the samples were in the range of 4–5 mg/mL. The diluted samples were then analyzed for ITF without exposure to thermal stress (native) or after ten minutes of thermal stress by dry block incubation. An aliquot of each diluted sample was incubated at 4°C, a second aliquot was incubated at 37°C, while a third aliquot was incubated at 80°C. Bovine serum albumin (BSA), diluted to 0.8 mg/mL, was included with the samples under each of the thermal conditions described above. All samples were re-equilibrated to room temperature after incubation. ITF Analysis was performed in triplicate by excitation at 280 nm with an emission scan range of 200–600 nm with a bandwidth of 1.0 nm, a Time-per point of 1 s, and a Step of 0.5. The triplicate spectra were blank-subtracted, averaged, and converted from units of mdeg to relative fluorescence intensity using MATLAB software. Diluted BSA solutions were assayed as controls preceding and following the sample sequence.

Phenotypic assessment of generated mature DCs by flow cytometry

To assess the expression of cell surface markers, *ex vivo* generated mature DCs were incubated with various antibodies diluted according to manufacturer's instructions using the staining buffer (PBS containing 2% FBS) for 30 min at 4°C in the dark. After extensive washing using the staining buffer, the cells were re-suspended in 400 μL of staining buffer and kept on ice until the signal was acquired using BD FACS Diva on CANTOII, then analyzed using FlowJoV10.

Monitoring antigen uptake and processing

To evaluate OVA uptake, mature DCs were first treated with 1 μg/mL of OVA-AF647 or Accum-OVA-AF647 for 1 hour at 37°C. Following their wash to remove excess antigen, the cells were incubated for 0.5, 1 and 3 hours prior to assessing their fluorescence by flow-cytometry. For evaluating antigen processing, mature DCs were incubated with 10 μg/mL OVA-DQ® (with or without Accum linking) at 37°C. Half an hour later, cells were washed, and regular media added. At the end of the indicated incubation time, cells were collected and washed with cold PBS containing 2% FBS. Fluorescence was monitored by flow cytometry. For antigen processing

experiments conducted using the proteasome inhibitors MG132 and lactacystin, the inhibitors were both used at a concentration of 10 μM on mature DCs for 1 hour at 37°C then washed prior to antigen pulsing.

Antigen cross-presentation assay

To evaluate antigen cross-presentation, cells were seeded at 25 × 10^3 cells per well in 24-well plate then pulsed with the antigens at different concentrations for 3 hours. At the end of the pulsing period, the cells were washed to remove excess antigen and co-cultured with 10^6/mL CD4 or CD8 T-cells purified from the spleen of OT-II or OT-I mouse, respectively, using T-cell isolation kits according to the manufacturer's protocol. Three days later, supernatants were collected and used to quantify cytokine production by commercial ELISAs. For the experiment using frozen cells, the same pulsing strategy was used followed by freezing mature DCs at −80°C for 30 days. The day of the experiment, cells were thawed, washed then plated directly with OT-I-derived CD8 T cells for three days. A similar approach was used to assess the effect of the proteasome inhibitors MG132 and lactacystin (both used at 10 μM) except that they were added to DCs 1 hour prior to washing and antigen pulsing.

For the B3Z assay, 5 × 10^4 mature DCs were first pulsed with the selected proteins for 3 hours followed by washing prior to adding 5 × 10^4 B3Z cells. The cells were incubated for 17–19 hours prior to their lysis and incubation for another 4–6 hours at 37°C with a CPRG solution. The optical density signal was detected at wavelength 570 using a SynergyH1 microplate reader (Biotek, Winooski, VT, United States).

Cancer cell lysate preparation

To prepare cancer cell lysates, cultured EL4 cells were collected by centrifugation at 1200 rpm for 5 min followed by two washing steps with PBS to remove traces of FBS. The cells were then subjected to 5 rounds of freeze and thaw cycles using liquid nitrogen/boiling water, respectively. To remove large particles, the lysate was shredded using a G26 needle, passed through a 70 μm cell strainer, then filtered with a 0.45 μm filter. The obtained lysate was then quantified using Bradford reagent, aliquoted and stored at −80°C until use.

Quantification of antibody titer by ELISA

The Nunc MaxiSorp^{TM} plates were coated overnight with 1μg nOVA diluted in coating buffer at 4°C. The following day, the plates were washed then blocked with 3% skim milk for 1 hour at room temperature. Following that step, the plates were washed prior to adding the diluted sera (two-fold dilutions were prepared). Following a 2-hour incubation period, the plates were washed prior to adding the secondary HRP-linked anti-mouse IgG antibody at a dilution of 1:1000. Two hours later, the plates were washed then incubated at room temperature with HRP for 10–20 min. Following HRP quenching, the signal was detected using a SynergyH1 microplate reader (Biotek, Winooski, VT, United States).

Analysis of tumor-infiltrating immune cells

Following their resection, tumor masses were first weighed then cut into smaller pieces with surgical scissors in 4-5 mL of master mix containing 2 mg/mL of Collagenase D, 2 mg/mL of collagenase IV, and 100 μg/mL of DNase type IV mixed in DMEM supplemented with 5% FBS. The mix was then stirred in a cell culture incubator at 37°C. After 30 min of incubation, 10 mL of DMEM was added to neutralize the enzymatic reaction. The digested solution was filtered using a 70 μm cell strainer and all retained fragments at the top of the strainer were smashed with a plunger followed by addition of 1-2 DMEM to wash the strainer. Collected cells were then centrifuges for 5 min at 1200 rpm (4°C), treated with a red blood cell lysis buffer for 1 min then resuspended in 3-4 mL of DMEM supplemented with 5% FBS. Following cell washing, the pellet was resuspended in DMEM supplemented with 5% FBS prior to initiate cell staining for flow cytometry analysis.

Modeling accessible lysine in protein antigen

Antigen 3D structure was modeled using the RCSB PDB and Swiss-Model Expasy free access software. Accessible amino acids representing the lysine residues were identified and highlighted according to their rate of accessibility (blue: high; green: medium and yellow: poor).

QUANTIFICATION AND STATISTICAL ANALYSIS

Statistical analysis

*p-value*s were calculated using the one-way analysis of variance (ANOVA) using GraphPad Prism. Results are represented as average mean with S.D. error bars, and statistical significance is represented with asterisks: *$P < 0.05$, **$P < 0.01$, ***$P < 0.001$.

Cell Reports Medicine, Volume *3*

Supplemental information

Promoting antigen escape from dendritic cell endosomes potentiates anti-tumoral immunity

Jean-Pierre Bikorimana, Natasha Salame, Simon Beaudoin, Mohammad Balood, Théo Crosson, Jamilah Abusarah, Sebastien Talbot, Raimar Löbenberg, Sebastien Plouffe, and Moutih Rafei

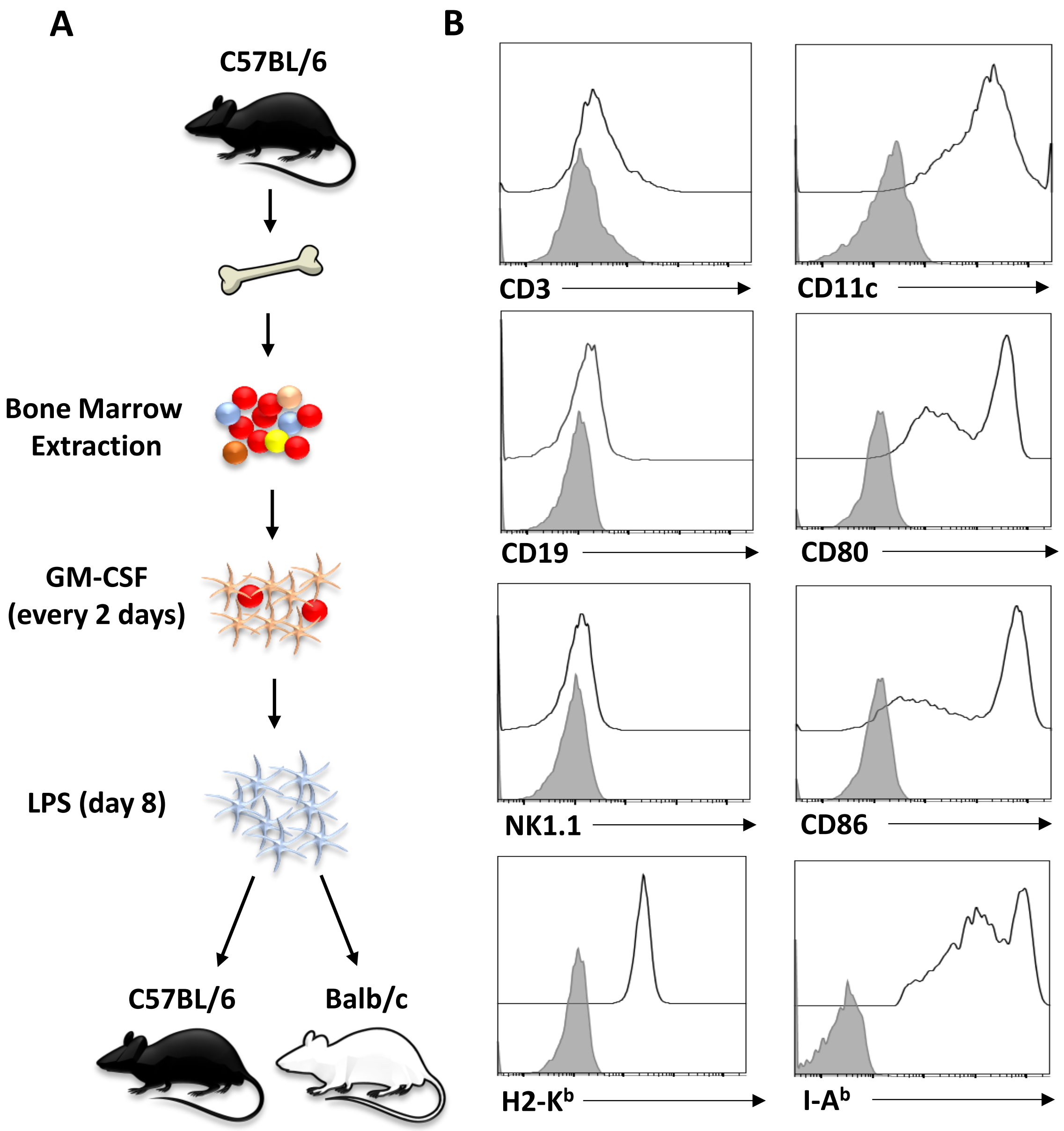

Supplementary figure 1: Generation and characterisation of BM-derived DC for vaccination. A) To generate BM-derived mature DCs, femur and tibias of female C57BL/6 or Balb/c mice are flushed to collect total nucleated cells. Cells are then plated for 8 days with recombinant GM-CSF (10 ng/ml) and replaced every 2 days. LPS is added on day 9 to trigger DC maturation prior to antigen pulsing. **B)** Representative flow-cytometry analysis of mature DC phenotype. No T cells, B cells or NK cells were detected at day 9. More than 80% of *ex vivo* generated mature DCs expressed $CD11c^{+}$, $CD80^{+}$, $CD86^{+}$, and I-A^{b+}. Related to figures 2, 4, 5 and 6.

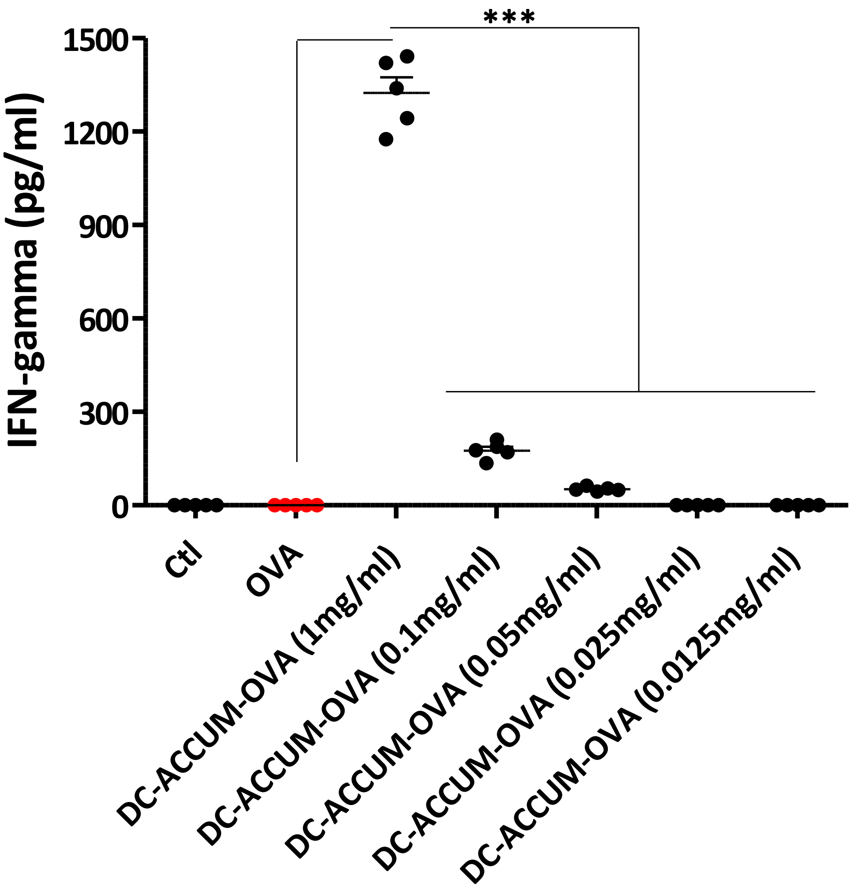

Supplementary figure 2: Quantification of IFN-gamma production by OT-II T cells. An antigen presentation assay was conducted using OT-II-derived CD4 T cells co-cultured with decreasing concentration of aOVA-pulsed mature DCs versus nOVA-pulsed mature DCs (shown in red). Three days following the co-culture, IFN-gamma was quantified using a commercial ELISA. For this panel, n=5/group with ***P<0.001. Related to figure 2.

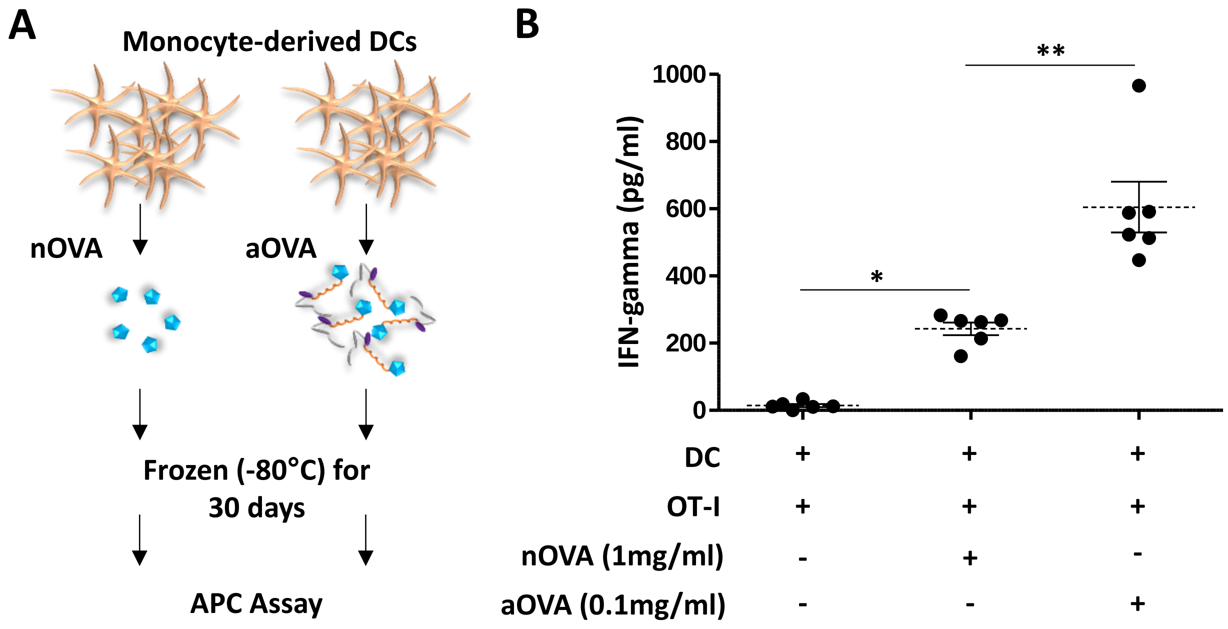

Supplementary figure 3: Frozen aOVA-pulsed mature DCs retain their potency to activate CD8 T cells. A) Representative cartoon of the experimental setting. **B)** Antigen presentation assay using frozen mature DCs originally pulsed with nOVA or aOVA. For this panel, n=6/group with *P<0.05 and **P<0.01. Related to figure 2.

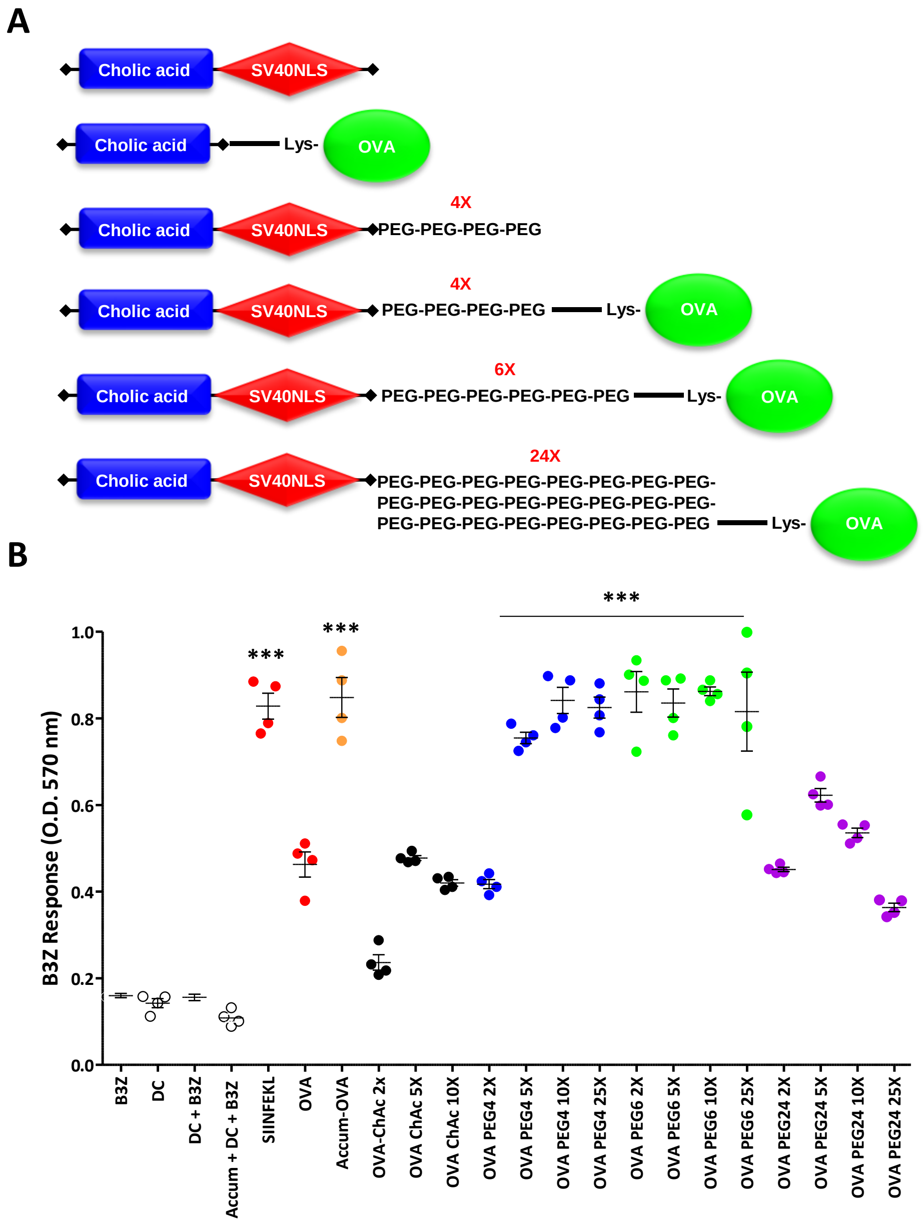

Supplementary figure 4: Testing the effect of various aOVA variants on the efficacy of antigen presentation by DCs. A) Representative cartoon of the various variants. The numbers 4X, 6X and 24X refers to the number of PEG molecules par Accum construct. The Accum-OVA is represented in the three-cartoon containing Cholic acid, SV40NLS, PEG and OVA. **B)** Response quantification using the SIINFEKL-specific B3Z cell line co-cultured with mature DCs treated with the different variants. The numbers 2X, 5X, 10X, and 25X refers to the number of Accum molecule per OVA. For this panel, n=5/group with ***P<0.001 when compared to the nOVA group. Related to figure 2.

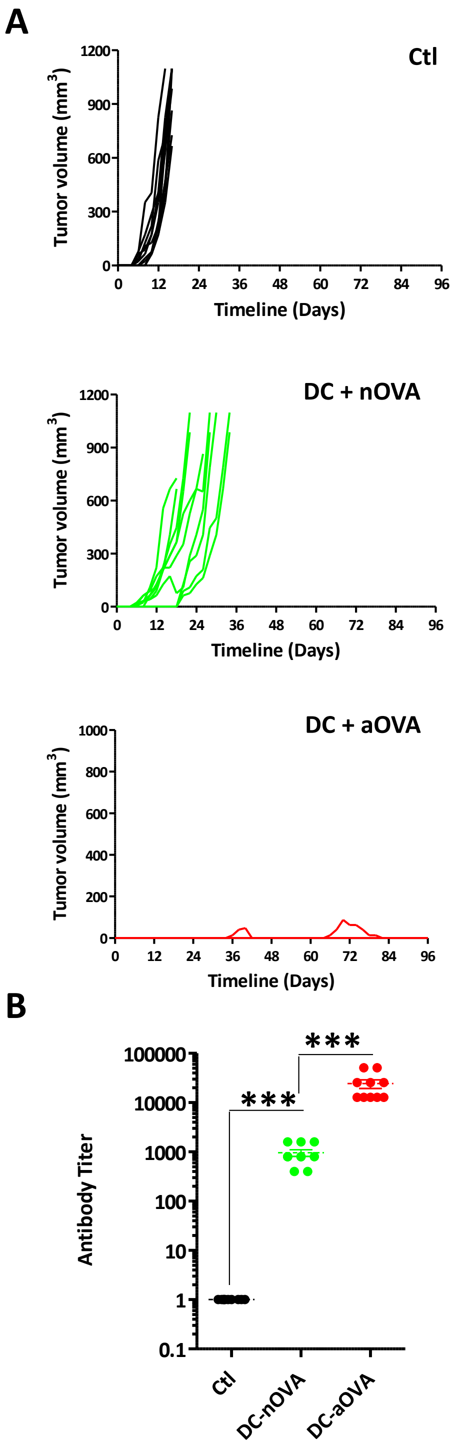

Supplementary figure 5: Individual tumor measurements and antibody tiers form the prophylactic vaccination experiment. A) Individual tumor measurements for EG.7 tumor growth in control mice (in black), OVA-pulsed mature DC-vaccinated mice (green) or Accum-OVA-pulsed mature DCs (red). **B)** Assessment of anti-OVA IgG titer by ELISA. For this experiment, n=10/group with ***P<0.001. Related to figure 4.

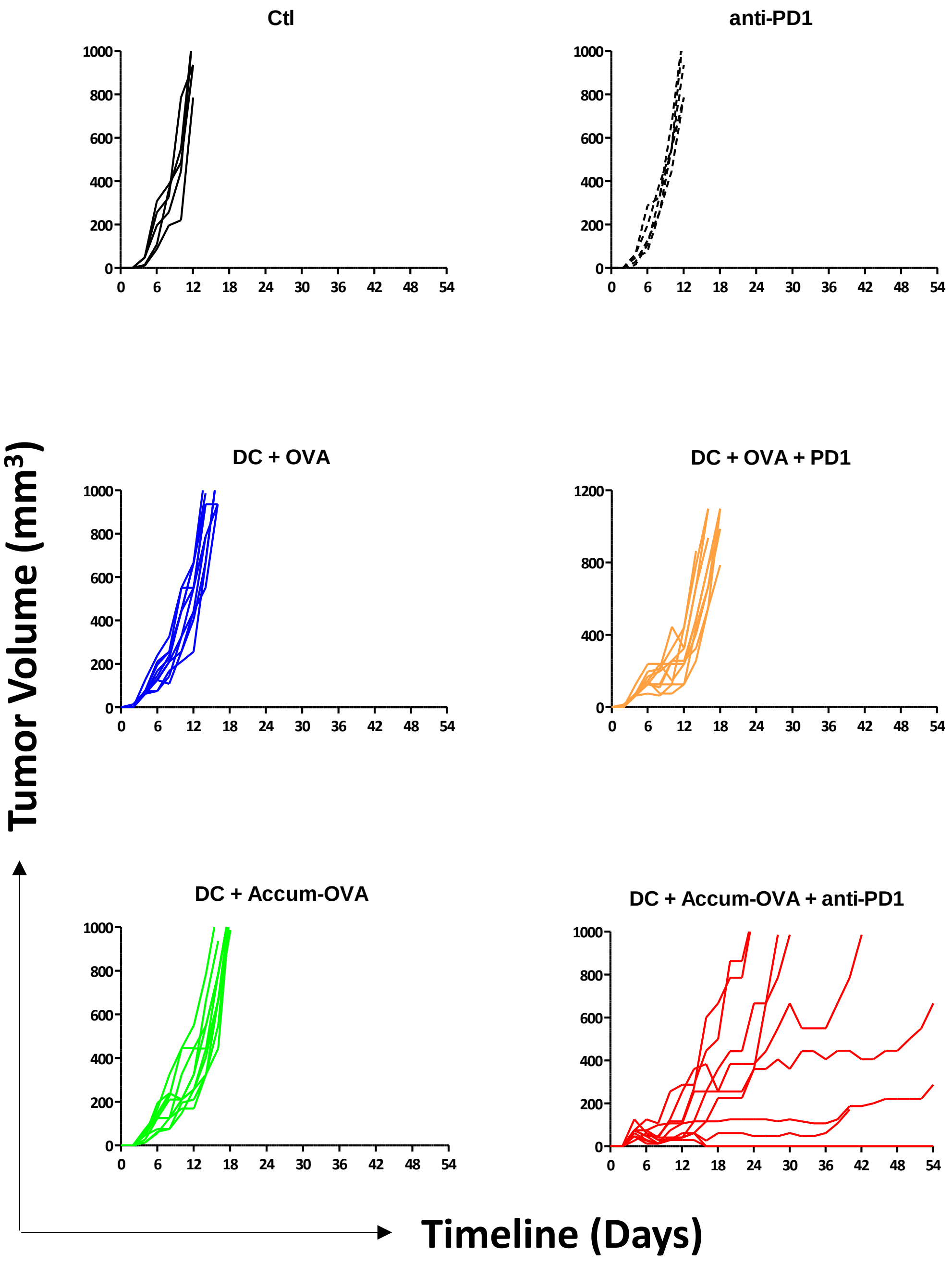

Supplementary figure 6: Individual tumor measurements from the syngeneic therapeutic vaccination study targeting EG.7 lymphoma. Individual tumor measurements for EG.7 tumor growth in control mice (in black), anti-PD-1-injected mice (black dotted lines), OVA-pulsed mature DC-vaccinated mice (blue), OVA-pulsed mature DC-vaccinated mice receiving anti-PD-1(orange), Accum-OVA-pulsed mature DCs (green), or Accum-OVA-pulsed mature DCs receiving anti-PD-1 (red). For this experiment, n=10/group. Related to figure 5.

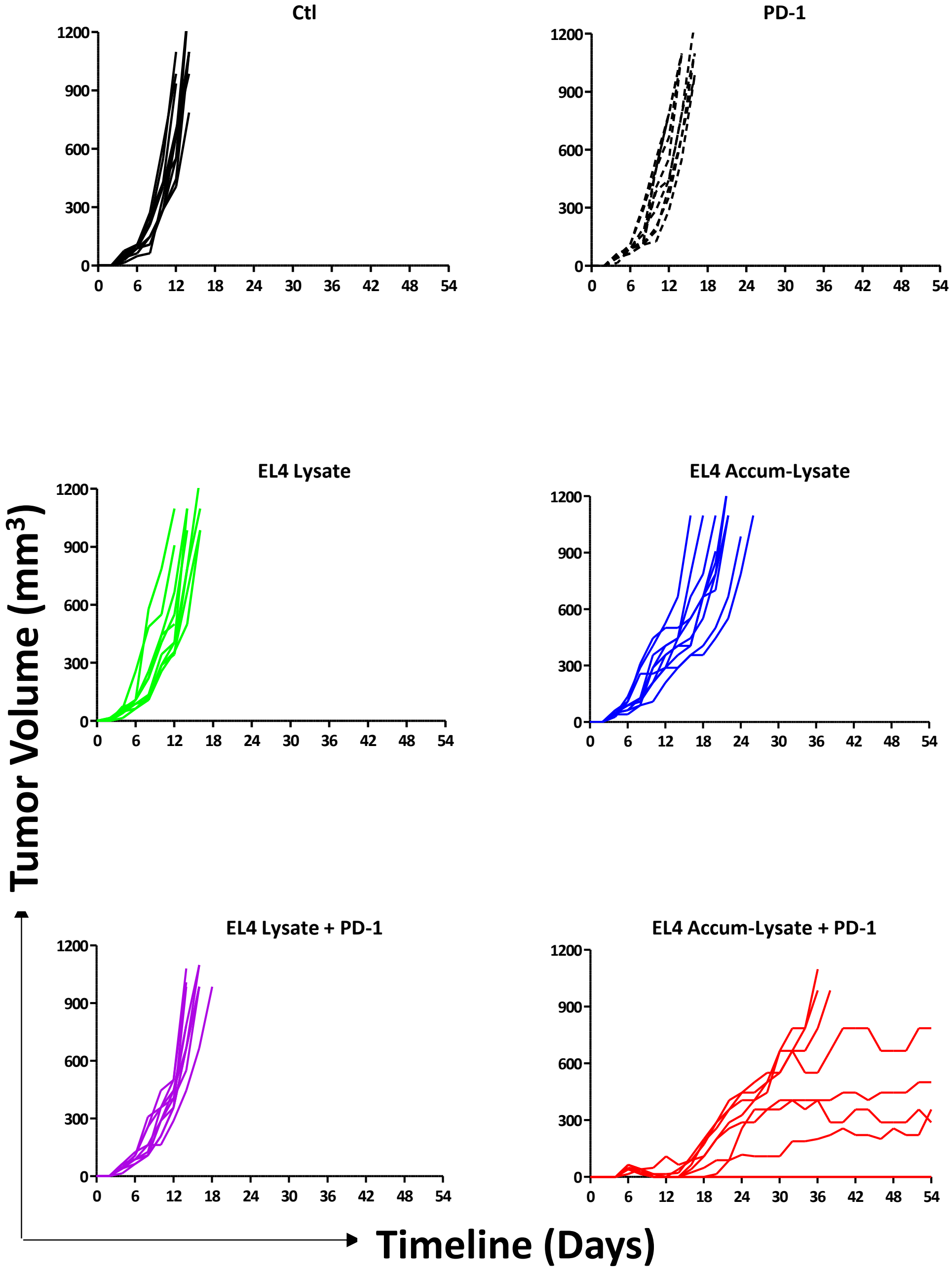

Supplementary figure 7: Individual tumor measurements from the allogeneic therapeutic vaccination targeting EL4 lymphoma. Individual tumor measurements for EL4 tumor growth in control mice (in black), anti-PD-1- injected mice (black dotted lines), EL4 lysate-pulsed mature DC-vaccinated mice (green), EL4 Accum-lysate-pulsed mature DC-vaccinated mice (blue), EL4 lysate-pulsed mature DC-vaccinated mice receiving anti-PD-1 (purple), or EL4 Accum-lysate-pulsed mature DC-vaccinated mice receiving anti-PD-1 (red). For this experiment, n=10/group. Related to figure 6.

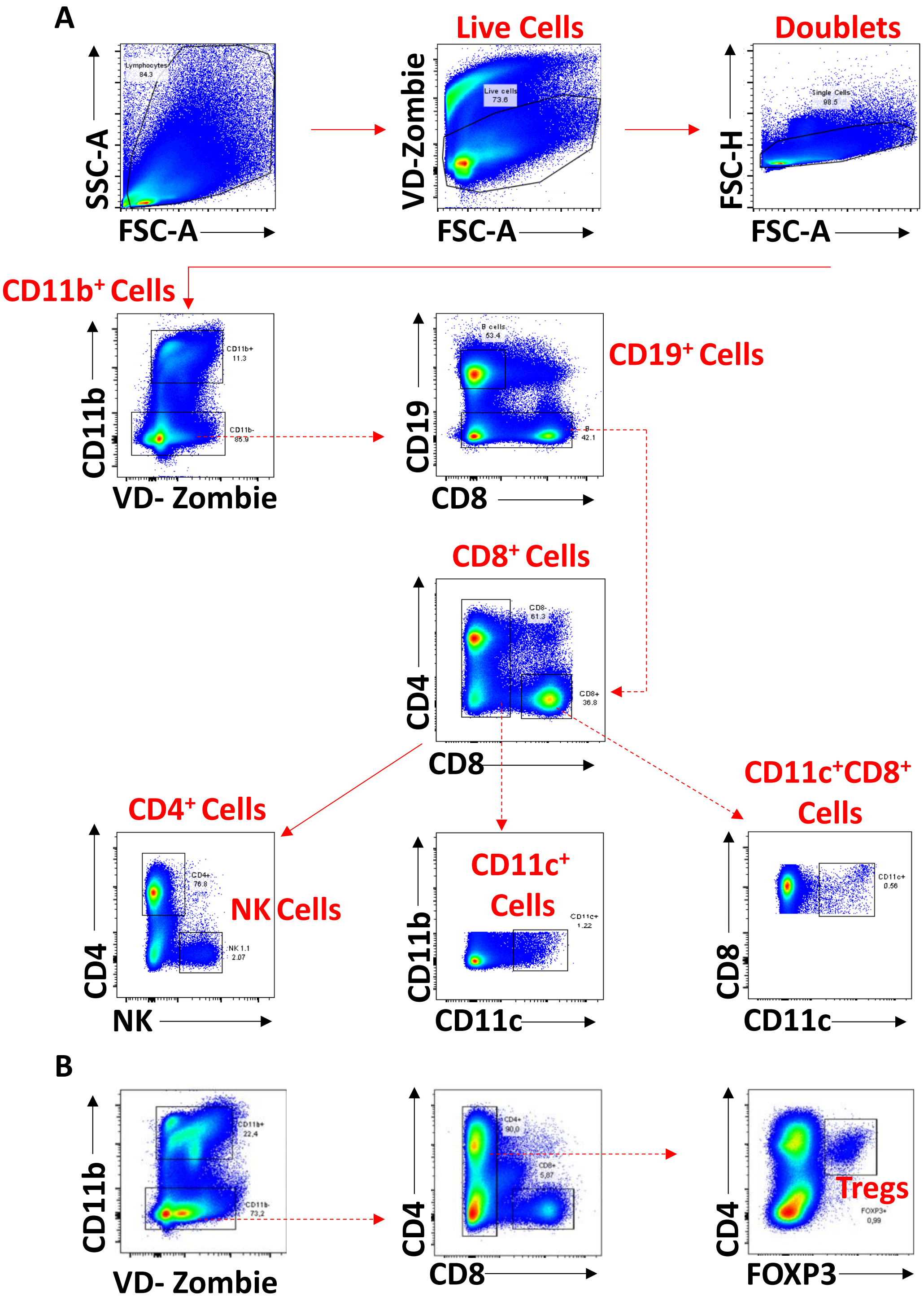

Supplementary figure 8: Gating strategies used for the TIL study. A) A representative gating strategy to demonstrate how various immune cells were identified. **B)** A representative gating strategy used to identify Tregs. Related to figure 6.
